# Predictors of hospital discharge and mortality in patients with diabetes and COVID-19: updated results from the nationwide CORONADO study

**DOI:** 10.1007/s00125-020-05351-w

**Published:** 2021-02-17

**Authors:** Matthieu Wargny, Louis Potier, Pierre Gourdy, Matthieu Pichelin, Coralie Amadou, Pierre-Yves Benhamou, Jean-Baptiste Bonnet, Lyse Bordier, Olivier Bourron, Claude Chaumeil, Nicolas Chevalier, Patrice Darmon, Blandine Delenne, Delphine Demarsy, Marie Dumas, Olivier Dupuy, Anna Flaus-Furmaniuk, Jean-François Gautier, Anne-Marie Guedj, Nathalie Jeandidier, Etienne Larger, Jean-Philippe Le Berre, Myriam Lungo, Nathanaëlle Montanier, Philippe Moulin, Françoise Plat, Vincent Rigalleau, René Robert, Dominique Seret-Bégué, Pierre Sérusclat, Sarra Smati, Jean-François Thébaut, Blandine Tramunt, Camille Vatier, Fritz-Line Velayoudom, Bruno Vergès, Patrice Winiszewski, Audrey Zabulon, Pierre-Antoine Gourraud, Ronan Roussel, Bertrand Cariou, Samy Hadjadj, for the CORONADO investigators

**Affiliations:** 1 l’institut du thorax, Inserm, CNRS, UNIV Nantes, CHU Nantes, Nantes, France; 2 CHU de Nantes, Inserm, CIC 1413, Pôle Hospitalo-Universitaire 11: Santé Publique, Clinique des Données, Nantes, France; 3 Département d’Endocrinologie, Diabétologie et Nutrition, Hôpital Bichat, Assistance Publique Hôpitaux de Paris, Paris, France; 4 Centre de Recherche des Cordeliers, Inserm, U-1138, Université de Paris, Paris, France; 5 Département d’Endocrinologie, Diabétologie et Nutrition, CHU Toulouse, Toulouse, France; 6 Institut des Maladies Métaboliques et Cardiovasculaires, UMR1048 Inserm/UPS, Université de Toulouse, Toulouse, France; 7 Département de Diabétologie, Centre Hospitalier Sud Francilien, Corbeil-Essonnes, France; 8 Université Paris-Saclay, Paris, France; 9 Service Endocrinologie-Diabétologie-Nutrition, CHU Grenoble, Grenoble, France; 10 University Grenoble-Alpes, Grenoble, France; 11 Département d’Endocrinologie, Diabète, Nutrition et CIC Inserm 1411, CHU de Montpellier, Montpellier, France; 12 Département de Diabétologie, H.I.A. Begin, Saint Mandé, France; 13 Sorbonne Université, Paris, France; 14 Assistance Publique Hôpitaux de Paris, Département de Diabétologie, CHU La Pitié Salpêtrière-Charles Foix, Paris, France; 15 Centre de Recherche des Cordeliers, Inserm, U-1138, Paris, France; 16 Institute of Cardiometabolism and Nutrition ICAN, Paris, France; 17 Fédération Française des Diabétiques (FFD), Paris, France; 18 Université Côte d’Azur, CHU, Inserm U1065, C3M, Nice, France; 19 Service d’Endocrinologie, Maladies Métaboliques et Nutrition, Hôpital de la Conception, Assistance Publique Hôpitaux de Marseille, Marseille, France; 20 C2VN, Inserm, INRA, Aix Marseille Université, Marseille, France; 21 Service d’Endocrinologie, Diabétologie et Maladies Métaboliques, Centre Hospitalier d’Aix-en-Provence, Aix-en-Provence, France; 22 Service Endocrinologie-Diabétologie, Centre Hospitalier de la Côte Basque, Bayonne, France; 23 Service Endocrinologie-Diabétologie, Hôpital Saint Vincent de Paul Lille, Lille, France; 24 Service de Diabétologie Endocrinologie, Hôpital Paris Saint-Joseph, Paris, France; 25 Service d’Endocrinologie - Diabétologie, Site Felix Guyon, CHU de la Réunion, Saint-Denis de la Réunion, France; 26 Service de Diabétologie et d’Endocrinologie, Hôpital Lariboisière, Assistance Publique Hôpitaux de Paris, Paris, France; 27 Service des Maladies Métaboliques et Endocriniennes, CHU de Nîmes, Université de Montpellier, Nîmes, France; 28 Service d’Endocrinologie, Diabétologie et Nutrition, Hôpitaux Universitaires de Strasbourg, UdS, Strasbourg, France; 29 Service de Diabétologie et Immunologie Clinique, Hôpital Cochin, Assistance Publique Hôpitaux de Paris, Centre-Université de Paris, Paris, France; 30 Service de Diabétologie et d’Endocrinologie, Médipôle, Villeurbanne, France; 31 Service d’Endocrinologie et de Diabétologie, Centre Hospitalier de Bastia, Bastia, France; 32 Service d’Endocrinologie, Centre Hospitalier du Forez, Montbrison, France; 33 Hôpital Cardiovasculaire Louis Pradel, Hospices Civils de Lyon, Inserm UMR 1060 Carmen, Université Claude Bernard Lyon 1, Lyon, France; 34 Service d’Endocrinologie et Maladies Métaboliques, Centre Hospitalier d’Avignon, Avignon, France; 35 Endocrinology-Nutrition Department, Centre Hospitalier Universitaire de Bordeaux, Université de Bordeaux, Bordeaux, France; 36 Université de Poitiers; CIC Inserm 1402; Médecine Intensive Réanimation, Centre Hospitalier Universitaire de Poitiers, Poitiers, France; 37 Service de Diabétologie, Endocrinologie et Nutrition, Centre Hospitalier de Gonesse, Gonesse, France; 38 Service d’Endocrinologie, Diabétologie et Maladies Métaboliques, Groupe Hospitalier Mutualiste Les Portes du Sud, Venissieux, France; 39 Assistance Publique Hôpitaux de Paris, Saint-Antoine Hospital, Reference Center of Rare Diseases of Insulin Secretion and Insulin Sensitivity (PRISIS), Department of Endocrinology, Paris, France; 40 Sorbonne University, Inserm UMRS 938, Saint-Antoine Research Center, Paris, France; 41 Service d’Endocrinologie, Diabétologie et Métabolisme, Centre Hospitalier Universitaire de Guadeloupe, Pointe-à-Pitre, France; 42 Service Endocrinologie, Diabétologie et Maladies Métaboliques, Hôpital du Bocage, Dijon, France; 43 Service d’Endocrinologie, Diabétologie et Nutrition, Hôpital Nord Franche-Comté, Trévenans, France; 44 Service d’Endocrinologie et Diabétologie, CHU de Martinique, Fort-de-France, France

**Keywords:** Admission plasma glucose, COVID-19, Death, Diabetes, Discharge, HbA_1c_, Home discharge, Mechanical ventilation

## Abstract

**Aims/hypothesis:**

This is an update of the results from the previous report of the CORONADO (Coronavirus SARS-CoV-2 and Diabetes Outcomes) study, which aims to describe the outcomes and prognostic factors in patients with diabetes hospitalised for coronavirus disease-2019 (COVID-19).

**Methods:**

The CORONADO initiative is a French nationwide multicentre study of patients with diabetes hospitalised for COVID-19 with a 28-day follow-up. The patients were screened after hospital admission from 10 March to 10 April 2020. We mainly focused on hospital discharge and death within 28 days.

**Results:**

We included 2796 participants: 63.7% men, mean age 69.7 ± 13.2 years, median BMI (25th–75th percentile) 28.4 (25.0–32.4) kg/m^2^. Microvascular and macrovascular diabetic complications were found in 44.2% and 38.6% of participants, respectively. Within 28 days, 1404 (50.2%; 95% CI 48.3%, 52.1%) were discharged from hospital with a median duration of hospital stay of 9 (5–14) days, while 577 participants died (20.6%; 95% CI 19.2%, 22.2%). In multivariable models, younger age, routine metformin therapy and longer symptom duration on admission were positively associated with discharge. History of microvascular complications, anticoagulant routine therapy, dyspnoea on admission, and higher aspartate aminotransferase, white cell count and C-reactive protein levels were associated with a reduced chance of discharge. Factors associated with death within 28 days mirrored those associated with discharge, and also included routine treatment by insulin and statin as deleterious factors.

**Conclusions/interpretation:**

In patients with diabetes hospitalised for COVID-19, we established prognostic factors for hospital discharge and death that could help clinicians in this pandemic period.

**Trial registration:**

Clinicaltrials.gov identifier: NCT04324736

**Graphical abstract:**

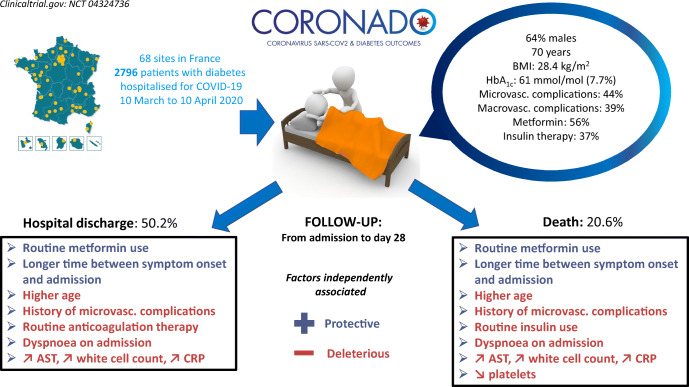

**Supplementary Information:**

The online version contains peer-reviewed but unedited supplementary material available at 10.1007/s00125-020-05351-w.

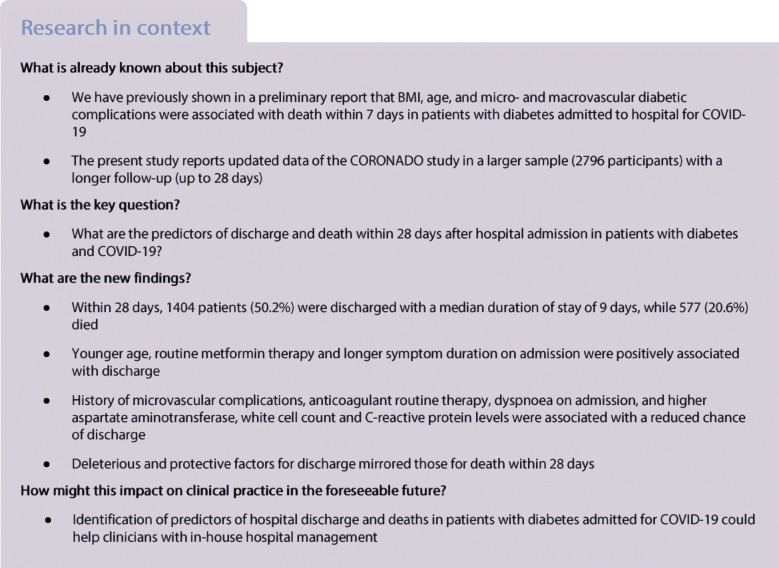



## Introduction

Soon after the coronavirus disease-2019 (COVID-19) pandemic outbreak, diabetes was rapidly identified as a risk factor for poor outcome [1, 2]. Diabetes has been previously recognised as a major risk factor for mortality in people infected with either the 2009 H1N1 pandemic influenza or the Middle East respiratory syndrome-related coronavirus (MERS-CoV) [3, 4]. More recently, diabetes consistently appeared as one of the major comorbidities associated with COVID-19 [1, 2, 5, 6].

Moreover, diabetes has been shown to be associated with worse prognosis. Indeed, the prevalence of diabetes was two- to threefold higher in patients admitted to intensive care units (ICUs) compared with those with less severe disease and the mortality rate was consistently higher in patients with diabetes [1, 2, 7–10]. Altogether, diabetes received considerable attention and was very often considered as a top risk factor for fatal COVID-19. In this context, contracting COVID-19 is a major source of worry in people with diabetes [11–13].

Diabetes-related worse COVID-19 prognosis has been extensively documented so far. In a short-term interim analysis of the CORONADO (Coronavirus-SARS-CoV-2 and Diabetes Outcomes) study, we showed that, in 1317 people with diabetes admitted for COVID-19, poor outcomes within 7 days were independently associated with BMI, age, diabetic complications, treated obstructive sleep apnoea (OSA), and some biological features [14]. However, to the best of our knowledge, no study has specifically focused on factors associated with favourable prognosis enabling hospital discharge in patients with diabetes admitted for COVID-19 infection.

Here, compared with our previous report, we expand our data to a larger sample size and a longer duration of follow-up. The current study describes the complete results of the CORONADO study, a French nationwide multicentre observational study aimed at identifying the clinical and biological features associated with disease severity and mortality in patients with diabetes hospitalised for COVID-19, with a specific focus on the clinical and biological characteristics of patients discharged home or to a long-term care facility by day 28.

## Methods

### Study design and participants

The design of the CORONADO study has been previously reported [14]. The aim of the study was to describe the phenotypic characteristics and prognosis of patients with diabetes presenting with COVID-19 between 10 March and 10 April 2020, in 68 French hospitals. Inclusion criteria were: (1) hospitalisation in a dedicated COVID-19 unit for biologically attested (PCR for severe acute respiratory syndrome coronavirus-2 [SARS-CoV-2]) and/or clinically/radiologically attested COVID-19 (i.e. as ground-glass opacity and/or crazy paving on chest computed tomography [CT] scan); (2) personal history of diabetes or newly diagnosed diabetes on admission (i.e. HbA_1c_ ≥6.5% [48 mmol/mol] during hospitalisation).

The study was designed in accordance with the Declaration of Helsinki and conducted in accordance with French legislation with approvals obtained from one local and two national committees: the local ethics committee (IRB/IEC – GNEDS; Ref. CORONADOV2), the CEREES (n° INDS:1544730) and the CNIL (DR-2020-155/920129). The latter waived informed consent owing to the purely observational approach using only retrospective data [14].

This article follows the Strengthening the Reporting of Observational Studies in Epidemiology (STROBE) reporting guidelines for cohort studies.

### Data collection

Data collection was retrospectively performed by clinical research associates and physicians in participating centres from medical files. They systematically reviewed the medical files of all COVID-19 inpatients, selected those with diabetes and, if necessary, contacted the patient’s general and/or specialist practitioners, regular pharmacist and biomedical laboratory. Collected data included clinical characteristics prior to admission including complications, comorbidities and routine treatments. Moreover, COVID-19-related clinical, radiological and biological characteristics were collected on admission. Regarding liver enzymes, the upper limit of normal was different between centres (see electronic supplementary material [ESM] Table 1).

### Patient follow-up and clinical outcomes

The composite primary outcome was combined tracheal intubation for mechanical ventilation and/or death within 7 days of admission. Participants discharged before day 7 were systematically contacted to check for the non-occurrence of these events on day 7.

Secondary outcomes included death, tracheal intubation, admission to ICUs and hospital discharge. A secondary time point was considered at day 28 for all patients alive and not discharged by day 7 in order to consider outcomes between admission and day 28. Outcomes were no longer collected after discharge on day 7 or later and no further follow-up was organised after day 28. Moreover, patients transferred to rehabilitation centres or to other hospitals were classified as ‘transferred’ and no follow-up information could be ascertained after the transfer. Those who remained in the hospital of their initial admission were classified as ‘in-hospital’. Only those returning home or to a long-term care facility were classified as ‘discharged’.

The current analysis mainly focused on hospital discharge and death within 28 days of follow-up.

### Statistical analyses

Quantitative data were expressed as mean ± SD or median (25th–75th percentile). Categorical variables were given as *n* (%) of participants. We described covariates according to time of admission, splitting data prior to and on admission, which corresponds to a clinician’s perspective. As specified in the protocol, the main objective of the study was descriptive. Originally, we calculated that, with a population of 300 participants and an attrition rate of 20%, an occurrence of 16% of the composite outcome would give a 95% CI equal to 11.7, 21.1% using the Clopper–Pearson estimate. In this analysis, we considered two main outcomes: hospital discharge and death within 28 days. The composite outcome (death and/or tracheal intubation) is only presented as ESM (ESM Tables 2–4).

Univariate logistic regression models were used to calculate ORs associated with study outcomes. Natural-log transformation was consistently considered in order to comply with linearity assumptions, which applied for diabetes duration, BMI and biological features.

Multivariable logistic regression models were used to assess the association between clinical and biological features and study outcomes (discharge and death within 28 days and primary outcome within 7 days). A standardisation process (*z* score) was also applied to quantitative variables to allow for direct comparison. Covariates included in the model were chosen according to a clinician’s point of view, based on background knowledge. The multivariate model included elementary clinical characteristics (sex, age, BMI); the patient’s history (hypertension, micro- and macrovascular complications, chronic obstructive pulmonary disease [COPD], treated OSA); routine medication (metformin, insulin, angiotensin-2 receptor blockers [ARBs] and/or ACE inhibitors and/or mineralocorticoid receptor antagonists [MRAs], statins, anticoagulation therapy); symptoms on admission (time between symptom onset and hospital admission, dyspnoea); and biological features (eGFR [determined using the CKD-EPI formula], aspartate aminotransferase [AST], white cell count, platelets, C-reactive protein [CRP]). Of note, the event-per-variable was always ≥10 in the main multivariable analyses.

Sensitivity analyses were performed for all multivariable models. First, we restricted the study population to the 2547 participants (91.1%) with a positive SARS-CoV-2 PCR. Second, we restricted the study population to the 1551 participants (55.5%) with admission plasma glucose data available.

All statistical tests were two-sided with a type 1 error set at 5%. All analyses were performed on available data, without imputation, and using statistical software R version 4.0.0 (https://cran.r-project.org).

## Results

### Study population and characteristics of COVID-19

The different centres originally screened 2951 patients suspected of hospitalisation or prolongation of hospitalisation related to COVID-19 between 10 March and 10 April 2020, with a diagnosis of diabetes mellitus (see flow diagram, ESM Fig. 1). After further investigations, 97 patients (3.3%) were ruled out for not meeting inclusion criteria (including 47 patients without diabetes mellitus and 35 without COVID-19). From the 2854 patients meeting inclusion criteria, 58 patients (2.0%) were excluded from the present analysis because of at least one unavailable key outcome, including seven with missing data on death during hospital stay and 25 with missing data on discharge. Finally, 2796 patients were analysed. Among those participants, 1317 (47.1%) were previously described in a recent interim analysis [14].

The baseline characteristics of the study participants are detailed in Tables 1 and 2. Briefly, participants were mostly male (63.7%), classified as having type 2 diabetes (88.2%), of Europid origin (58.1%), and with a mean ± SD age of 69.7 ± 13.2 years, a median (25th–75th percentile) BMI of 28.4 (25.0–32.4) kg/m^2^ and a median (25th–75th percentile) HbA_1c_ value of 60.7 mmol/mol (50.8–74.9 mmol/mol) (7.7% [6.8–9.0%]). On admission, a positive SARS-CoV-2 PCR was found in 2547/2705 (94.2%) patients and median (25th–75th percentile) CRP was 86 (40–148) mg/l.

**Table 1:** Clinical characteristics prior to admission of CORONADO participants according to the discharge within 28 days

			Discharge within 28 days				
Clinical features	Available data	All (*N* = 2796)	No (*n* = 1392)	Yes (*n* = 1404)	Unadjusted OR (95% CI)	Age-adjusted OR (95% CI)	Age-adjusted *p* value
Sex (female)	2796	1014/2796 (36.3)	467/1392 (33.5)	547/1404 (39.0)	1.26 (1.08, 1.48)	1.38 (1.18, 1.62)	<0.001
Age (years)	2796	69.7 ± 13.2	73.1 ± 12.4	66.3 ± 13.1	0.96 (0.95, 0.97)	–	<0.001
Age class (years)	2796						<0.001
<55		367/2796 (13.1)	117/1392 (8.4)	250/1404 (17.8)	1		
55–64		565/2796 (20.2)	209/1392 (15.0)	356/1404 (25.4)	0.80 (0.60, 1.05)	–	0.11
65–74		797/2796 (28.5)	394/1392 (28.3)	403/1404 (28.7)	0.48 (0.37, 0.62)	–	<0.001
≥75		1067/2796 (38.2)	672/1392 (48.3)	395/1404 (28.1)	0.28 (0.21, 0.35)	–	<0.001
Ethnicity	2384						0.13
EU		1385/2384 (58.1)	745/1193 (62.4)	640/1191 (53.7)	1	1	
MENA		497/2384 (20.8)	227/1193 (19.0)	270/1191 (22.7)	1.38 (1.13, 1.70)	1.17 (0.95, 1.45)	0.15
AC		415/2384 (17.4)	174/1193 (14.6)	241/1191 (20.2)	1.61 (1.29, 2.01)	1.06 (0.83, 1.34)	0.65
AS		87/2384 (3.6)	47/1193 (3.9)	40/1191 (3.4)	0.99 (0.64, 1.53)	0.68 (0.43, 1.08)	0.10
BMI (kg/m^2^)	2460	28.4 (25–32.4)	28.2 (24.8–32.2)	28.7 (25.3–32.5)	1.09 (1.01, 1.18)	0.99 (0.91, 1.07)	0.77
BMI class	2460						0.66
<25 kg/m^2^		611/2460 (24.8)	325/1226 (26.5)	286/1234 (23.2)	1	1	
25–29.9 kg/m^2^		890/2460 (36.2)	437/1226 (35.6)	453/1234 (36.7)	1.18 (0.96, 1.45)	1.09 (0.88, 1.35)	0.42
30–39.9 kg/m^2^		823/2460 (33.5)	398/1226 (32.5)	425/1234 (34.4)	1.21 (0.98, 1.50)	1.00 (0.80, 1.25)	0.99
≥40 kg/m^2^		136/2460 (5.5)	66/1226 (5.4)	70/1234 (5.7)	1.21 (0.83, 1.75)	0.90 (0.61, 1.32)	0.58
Diabetes duration (years)	1767	11 (5–20)	12 (5–20)	10 (4–18)	0.84 (0.76, 0.92)	0.99 (0.90, 1.10)	0.92
HbA_1c_ (mmol/mol)^a^	1800	60.7 (50.8–74.9)	59.6 (50.8–72.7)	61.8 (50.8–77.1)	1.19 (1.08, 1.31)	1.06 (0.96, 1.17)	0.24
HbA_1c_ (%)^a^	1800	7.7 (6.8–9.0)	7.6 (6.8–8.8)	7.8 (6.8–9.2)	1.19 (1.08, 1.31)	1.06 (0.96, 1.17)	0.24
Hypertension	2769	2126/2769 (76.8)	1122/1379 (81.4)	1004/1390 (72.2)	0.60 (0.50, 0.71)	0.80 (0.66, 0.97)	0.025
Dyslipidaemia	2710	1267/2710 (46.8)	661/1342 (49.3)	606/1368 (44.3)	0.82 (0.70, 0.95)	0.93 (0.79, 1.08)	0.35
Current tobacco use	2288	131/2288 (5.7)	63/1109 (5.7)	68/1179 (5.8)	1.02 (0.71, 1.45)	0.93 (0.65, 1.34)	0.69
Long-term diabetes complications							
Microvascular complications^b^	1966	869/1966 (44.2)	516/939 (55.0)	353/1027 (34.4)	0.43 (0.36, 0.52)	0.59 (0.49, 0.72)	<0.001
Macrovascular complications^c^	2627	1014/2627 (38.6)	586/1297 (45.2)	428/1330 (32.2)	0.58 (0.49, 0.67)	0.74 (0.63, 0.88)	<0.001
Comorbidities							
Heart failure	2651	302/2651 (11.4)	190/1308 (14.5)	112/1343 (8.3)	0.54 (0.42, 0.69)	0.71 (0.55, 0.92)	0.009
NAFLD or liver cirrhosis	2640	218/2640 (8.3)	102/1310 (7.8)	116/1330 (8.7)	1.13 (0.86, 1.49)	0.98 (0.73, 1.30)	0.87
Active cancer	2742	253/2742 (9.2)	137/1362 (10.1)	116/1380 (8.4)	0.82 (0.63, 1.06)	1.05 (0.80, 1.37)	0.73
COPD	2732	263/2732 (9.6)	162/1359 (11.9)	101/1373 (7.4)	0.59 (0.45, 0.76)	0.70 (0.54, 0.92)	0.009
Treated OSA	2593	273/2593 (10.5)	144/1284 (11.2)	129/1309 (9.9)	0.87 (0.67, 1.11)	0.84 (0.65, 1.09)	0.20
Routine treatment before admission							
Metformin	2794	1553/2794 (55.6)	690/1391 (49.6)	863/1403 (61.5)	1.62 (1.40, 1.89)	1.46 (1.25, 1.71)	<0.001
Sulfonylurea/glinides	2794	782/2794 (28.0)	378/1391 (27.2)	404/1403 (28.8)	1.08 (0.92, 1.28)	1.13 (0.96, 1.34)	0.15
DPP4-inhibitors	2794	615/2794 (22.0)	286/1391 (20.6)	329/1403 (23.4)	1.18 (0.99, 1.42)	1.22 (1.02, 1.47)	0.033
GLP-1RA	2794	254/2794 (9.1)	108/1391 (7.8)	146/1403 (10.4)	1.38 (1.06, 1.79)	1.11 (0.85, 1.45)	0.45
Insulin	2796	1039/2796 (37.2)	563/1392 (40.4)	476/1404 (33.9)	0.76 (0.65, 0.88)	0.78 (0.67, 0.92)	0.002
Diuretics^d^	2794	1058/2794 (37.9)	602/1391 (43.3)	456/1403 (32.5)	0.63 (0.54, 0.74)	0.78 (0.66, 0.91)	0.002
β-Blockers	2794	988/2794 (35.4)	552/1391 (39.7)	436/1403 (31.1)	0.69 (0.59, 0.80)	0.84 (0.71, 0.99)	0.033
CCB	2794	924/2794 (33.1)	487/1391 (35.0)	437/1403 (31.1)	0.84 (0.72, 0.98)	0.91 (0.77, 1.07)	0.25
ARBs and/or ACE inhibitors and/or MRA^e^	2794	1570/2794 (56.2)	800/1391 (57.5)	770/1403 (54.9)	0.90 (0.77, 1.04)	1.03 (0.88, 1.20)	0.75
Statins	2794	1282/2794 (45.9)	664/1391 (47.7)	618/1403 (44.0)	0.86 (0.74, 1.00)	0.96 (0.82, 1.12)	0.63
Anti-platelet therapy	2794	1125/2794 (40.3)	594/1391 (42.7)	531/1403 (37.8)	0.82 (0.70, 0.95)	1.03 (0.88, 1.20)	0.75
Anticoagulation therapy	2794	501/2794 (17.9)	321/1391 (23.1)	180/1403 (12.8)	0.49 (0.40, 0.60)	0.65 (0.53, 0.80)	<0.001

Population size was *N* = 2796. Data shown are *n* (%), and mean ± SD or median (25th–75th percentile) if not normally distributed

*p* values are calculated using Wald test (unadjusted and age-adjusted logistic regression, except for ‘age’ and ‘age class’, which were not adjusted). ORs correspond to an increase of 1 SD after natural-log transformation and standardisation for BMI, diabetes duration and HbA_1c_

^a^HbA_1c_ determined in the 6 months prior to or in the first 7 days following hospital admission

^b^Microvascular complication was defined as history of one or more of the following: diabetic kidney disease and/or severe diabetic retinopathy and/or diabetic foot ulcer

^c^Macrovascular complication was defined as history of one or more of the following: ischaemic heart disease (acute coronary syndrome and/or coronary artery revascularisation) and/or cerebrovascular disease (stroke and/or transient ischaemic attack) and/or peripheral artery disease (amputation owing to ischaemic disease and/or lower limb artery revascularisation)

^d^Diuretics include loop diuretics, thiazide diuretics and potassium-sparing diuretics

^e^MRAs include spironolactone and eplerenone

Abbreviations: EU, Europid; MENA, Middle East North Africa; AC, African or Caribbean; AS, Asian; NAFLD, non-alcoholic fatty liver disease; GLP-1RA, glucagon-like peptide 1-receptor agonist

**Table 2:** Characteristics on admission of CORONADO participants, according to discharge within 28 days

			Discharge within 28 days				
Variable	Available data	All (*N* = 2796)	No (*n* = 1392)	Yes (*n* = 1404)	Unadjusted OR (95% CI)	Age-adjusted OR (95% CI)	Age-adjusted *p* value
Time from symptom onset to hospital admission (days)	2743	5 (2–9)	4 (2–7)	6 (3–9)	1.05 (1.04, 1.07)	1.04 (1.02, 1.05)	<0.001
Clinical presentation							
Fever	2755	2077/2755 (75.4)	1031/1364 (75.6)	1046/1391 (75.2)	0.98 (0.82, 1.16)	0.85 (0.71, 1.01)	0.068
Dyspnoea	2754	1771/2754 (64.3)	942/1364 (69.1)	829/1390 (59.6)	0.66 (0.57, 0.77)	0.62 (0.52, 0.73)	<0.001
Abnormal chest CT scan	1982	1919/1982 (96.8)	898/927 (96.9)	1021/1055 (96.8)	0.97 (0.59, 1.60)	0.83 (0.49, 1.39)	0.48
Biological findings							
Positive SARS-CoV-2 PCR	2705	2547/2705 (94.2)	1286/1340 (96.0)	1261/1365 (92.4)	0.51 (0.36, 0.71)	0.46 (0.33, 0.66)	<0.001
Admission plasma glucose (mmol/l)	1551	9.5 (7.0–13.5)	10.0 (7.1–14.0)	9.1 (6.9–12.8)	0.89 (0.80, 0.98)	0.82 (0.73, 0.91)	<0.001
Plasma creatinine (μmol/l)	2602	91 (69–133)	103 (75–153)	84 (65–115)	0.69 (0.63, 0.75)	0.73 (0.67, 0.80)	<0.001
eGFR (ml min^−1^ [1.73 m]^−2^)^a^	2602	68.5 (41.8–89.7)	57.1 (34.5–82.7)	78.5 (52.0–95.6)	1.57 (1.44, 1.71)	1.36 (1.25, 1.49)	<0.001
ALT (%ULN)	2478	0.62 (0.42–1.00)	0.62 (0.40–0.99)	0.62 (0.43–1.00)	0.96 (0.89, 1.04)	0.87 (0.80, 0.95)	0.002
AST (%ULN)	2444	1.06 (0.75–1.60)	1.17 (0.80–1.85)	0.98 (0.70–1.38)	0.68 (0.62, 0.74)	0.64 (0.58, 0.71)	<0.001
GGT (%ULN)	2317	0.95 (0.55–1.77)	0.99 (0.56–1.88)	0.92 (0.55–1.63)	0.89 (0.82, 0.96)	0.85 (0.78, 0.92)	<0.001
Haemoglobin (g/l)	2728	127 (114–142)	125 (110–141)	129 (118–143)	1.28 (1.18, 1.38)	1.21 (1.12, 1.31)	<0.001
White cell count (10^3^/mm^3^)	2726	6580 (5000–8818)	6800 (5150–9370)	6310 (4900–8400)	0.82 (0.76, 0.88)	0.82 (0.76, 0.89)	<0.001
Lymphocyte count (10^3^/mm^3^)	2646	990 (690–1400)	900 (600–1300)	1100 (780–1470)	1.29 (1.19, 1.40)	1.23 (1.13, 1.33)	<0.001
Platelet count (10^3^/mm^3^)	2725	201 (156–260)	196 (150–254)	205 (164–264)	1.17 (1.08, 1.26)	1.13 (1.04, 1.22)	0.003
CRP (mg/l)	2612	86.0 (40.3–148.0)	98.6 (49.2–166.5)	73.0 (33.4–128.0)	0.74 (0.68, 0.80)	0.70 (0.65, 0.76)	<0.001
LDH (U/l)	1427	351 (266–498)	402 (290–567)	319 (250–419)	0.60 (0.52, 0.70)	0.56 (0.48, 0.66)	<0.001
CPK (U/l)	1385	132 (67–305)	164 (72–416)	108 (61–222)	0.70 (0.62, 0.78)	0.67 (0.60, 0.75)	<0.001
Fibrinogen (g/l)	1424	6.3 (5.0–7.4)	6.3 (5.0–7.6)	6.2 (5.0–7.3)	0.99 (0.89, 1.10)	0.96 (0.87, 1.07)	0.51

Population size was *N* = 2796. Data shown are *n* (%) and or median (25th–75th percentile)

*p* values are calculated using Wald test (unadjusted and age-adjusted logistic regression). Quantitative variables were natural-log transformed and associated ORs correspond to an increase of 1 SD after standardisation, except for time from symptoms onset to hospital admission (1 day increase)

^a^eGFR determined by the CKD-EPI formula

Abbreviations: ALT, alanine aminotransferase; ULN, upper limit of normal; GGT, γ-glutamyl transferase; LDH, lactate dehydrogenase; CPK, creatine phosphokinase

**Fig. 1:**
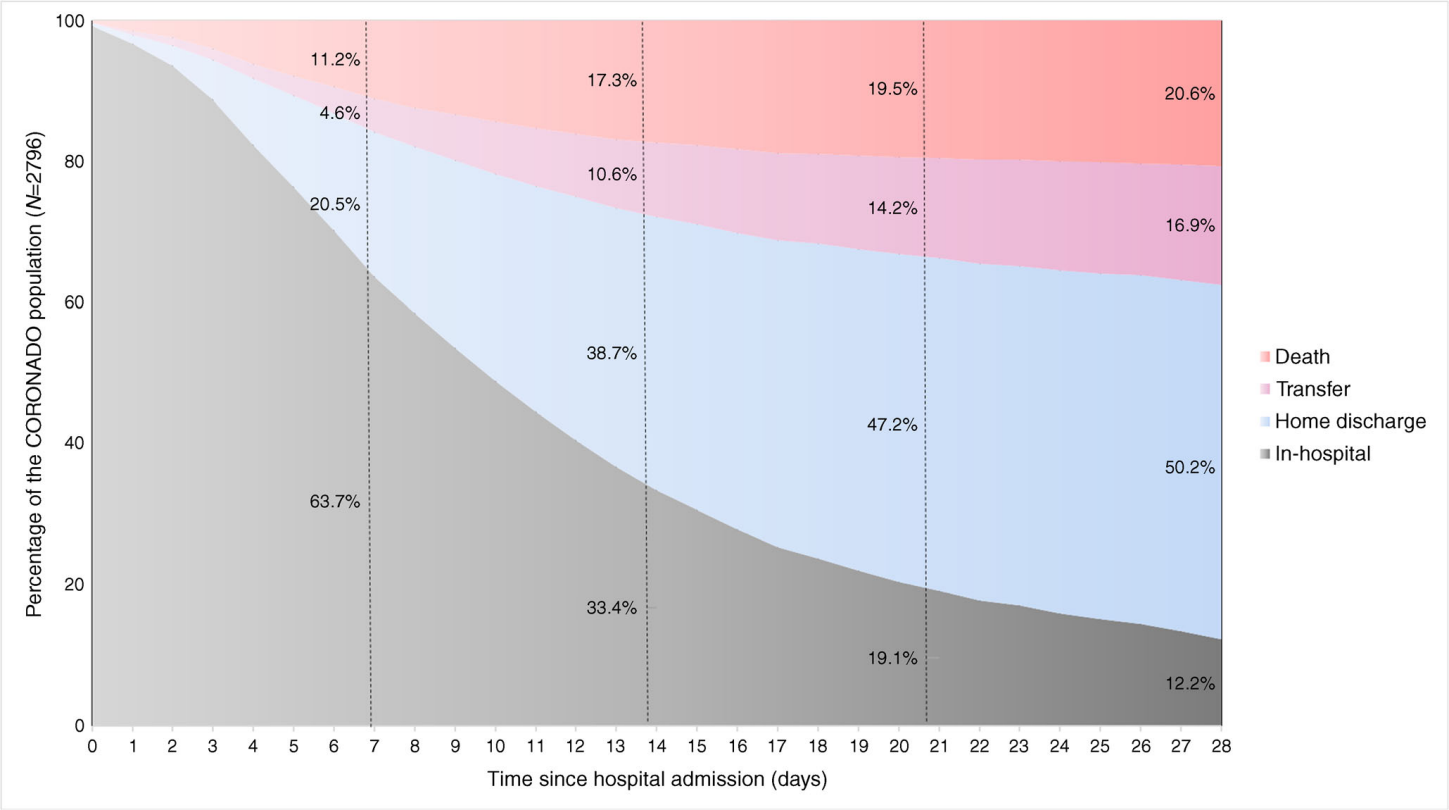
Distribution of patient outcomes according to time since hospital admission. Population size was *N* = 2796

COVID-19-related clinical outcomes are detailed in ESM Table 5 and the timeframe for study outcomes is presented in Fig. 1.

### Discharge within 28 days

As shown in Fig. 1, 50.2% of the CORONADO participants were discharged within 28 days. Their median (25th–75th percentile) length of stay before discharge was 9 (5–14) days with 156/1404 (11.1%) discharged in the first 3 days following admission. Since the management of COVID-19 has been rapidly evolving, we analysed the length of stay according to three consecutive admission periods (see ESM Fig. 2a,b) and highlighted no statistical difference, with 18/234 (7.7%), 104/886 (11.7%) and 34/284 (12.0%) patients discharged in the first 3 days who were admitted 10–19 March, 20–30 March, and 31 March to 10 April, respectively (*p* = 0.18).

The characteristics of the participants discharged by day 28 are presented in Table 1 and Table 2. Briefly, women were more likely than men to be discharged. Older age was associated with a lower chance of discharge (for a 1-year increment, OR 0.96; 95% CI 0.95, 0.97). Hypertension, micro- or macrovascular diabetic complications, heart failure and COPD were also associated with a lower chance of discharge. Regarding routine therapy, metformin and dipeptidyl peptidase 4 (DPP-4) inhibitors were associated with a higher chance of discharge, whereas insulin, diuretics, β-blockers and anticoagulation therapy were associated with a lower chance.

As regards patient characteristics on admission, dyspnoea, plasma creatinine, and almost all biological findings associated with an inflammatory profile (including higher AST, higher white cell and lower lymphocyte cell count, and higher CRP) were significantly associated with a lower chance of discharge.

**Fig. 2:**
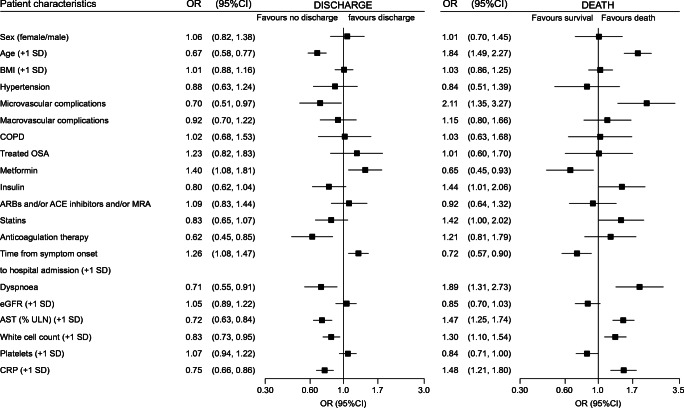
Multivariable analysis of hospital discharge and death within 28 days: covariates prior to and on admission. Models were applied to 1355 participants yielding 728 discharges (53.7%) and 225 deaths within 28 days (16.6%). Regarding quantitative variables: all were natural-log transformed, except for age and BMI, and the ORs correspond to an increase of 1 SD after standardisation. The model on the left uses discharge within 28 days as the dependent variable, and the model on the right uses death within 28 days. Microvascular complication was defined as history of one or more of the following: diabetic kidney disease and/or severe diabetic retinopathy and/or history of diabetic foot ulcer. Macrovascular complication was defined as history of one or more of the following: ischaemic heart disease (acute coronary syndrome and/or coronary artery revascularisation) and/or cerebrovascular disease (stroke and/or transient ischaemic attack) and/or peripheral artery disease (amputation owing to ischaemic disease and/or lower limb artery revascularisation) MRAs include spironolactone and eplerenone; eGFR was determined by the CKD-EPI formula. ULN, upper limit of normal

The results of multivariable analysis of discharge within 28 days are presented in Fig. 2 (see ESM Table 6 for results of the raw multivariable analysis, without transformation). Predictors of discharge on day 28 were younger age, routine metformin therapy and longer time between symptom onset and hospital admission. Conversely, history of microvascular complications, routine anticoagulant therapy, dyspnoea, higher AST, white cell count and CRP levels were associated with a reduced chance of discharge.

In the sensitivity analysis on the complete-case population restricted to the patients with positive SARS-CoV-2 PCR (*n* = 1249) or admission plasma glucose available and added to the model (*n* = 761), using the same variables as those used in the multivariable model applied to the whole population, we found very similar results (ESM Table 7).

### Death within 28 days

Characteristics prior to admission of those patients who died within 28 days are presented in Table 3. In age-adjusted analysis, male sex, longer duration of diabetes, as well as history of micro- and macrovascular complications, heart failure and COPD were significantly associated with death.

**Table 3:** Clinical characteristics prior to admission of CORONADO participants, according to death within 28 days

		Death within 28 days				
Clinical features	Available data	No (*n* = 2219)	Yes (*n* = 577)	Unadjusted OR (95% CI)	Age-adjusted OR (95% CI)	Age-adjusted *p* value
Sex (female)	2796	826/2219 (37.2)	188/577 (32.6)	0.82 (0.67, 0.99)	0.69 (0.56, 0.84)	<0.001
Age (years)	2796	67.9 ± 13.0	76.8 ± 11.3	1.06 (1.05, 1.07)	–	<0.001
Age class (years)	2796					<0.001
<55		345/2219 (15.5)	22/577 (3.8)	1		
55–64		511/2219 (23.0)	54/577 (9.4)	1.66 (0.99, 2.77)	–	0.054
65–74		657/2219 (29.6)	140/577 (24.3)	3.34 (2.09, 5.34)	–	<0.001
≥75		706/2219 (31.8)	361/577 (62.6)	8.02 (5.12, 12.6)	–	<0.001
Ethnicity	2384					0.35
EU		1065/1895 (56.2)	320/489 (65.4)	1	1	
MENA		406/1895 (21.4)	91/489 (18.6)	0.75 (0.58, 0.97)	0.98 (0.74, 1.28)	0.86
AC		357/1895 (18.8)	58/489 (11.9)	0.54 (0.40, 0.73)	1.01 (0.73, 1.41)	0.94
AS		67/1895 (3.5)	20/489 (4.1)	0.99 (0.59, 1.66)	1.67 (0.96, 2.88)	0.068
BMI (kg/m^2^)	2460	28.6 (25.2–32.6)	27.8 (24.5–31.7)	0.91 (0.82, 1.00)	1.06 (0.95, 1.18)	0.31
BMI class	2460					0.17
<25 kg/m^2^		473/1973 (24.0)	138/487 (28.3)	1	1	
25–29.9 kg/m^2^		716/1973 (36.3)	174/487 (35.7)	0.83 (0.65, 1.07)	0.96 (0.74, 1.25)	0.78
30–39.9 kg/m^2^		679/1973 (34.4)	144/487 (29.6)	0.73 (0.56, 0.94)	0.98 (0.74, 1.29)	0.89
≥40 kg/m^2^		105/1973 (5.3)	31/487 (6.4)	1.01 (0.65, 1.58)	1.63 (1.02, 2.60)	0.042
Diabetes duration (years)	1767	10 (4–18)	15 (8–23)	1.47 (1.27, 1.69)	1.20 (1.04, 1.39)	0.015
HbA_1c_ (mmol/mol)^a^	1800	61.6 (50.8–76.0)	59.6 (50.8–69.4)	0.80 (0.70, 0.91)	0.90 (0.78, 1.04)	0.16
HbA_1c_ (%)^a^	1800	7.8 (6.8–9.1)	7.6 (6.8–8.5)	0.80 (0.70, 0.91)	0.90 (0.78, 1.04)	0.16
Hypertension	2769	1644/2198 (74.8)	482/571 (84.4)	1.83 (1.43, 2.33)	1.25 (0.97, 1.62)	0.090
Dyslipidaemia	2710	981/2157 (45.5)	286/553 (51.7)	1.28 (1.06, 1.55)	1.15 (0.95, 1.40)	0.16
Current tobacco use	2288	109/1855 (5.9)	22/433 (5.1)	0.86 (0.54, 1.37)	1.00 (0.61, 1.62)	0.99
Long-term diabetes complications						
Microvascular complications^b^	1966	604/1591 (38.0)	265/375 (70.7)	3.94 (3.08, 5.03)	2.63 (2.03, 3.40)	<0.001
Macrovascular complications^c^	2627	725/2089 (34.7)	289/538 (53.7)	2.18 (1.80, 2.65)	1.60 (1.30, 1.95)	<0.001
Comorbidities						
Heart failure	2651	200/2121 (9.4)	102/530 (19.2)	2.29 (1.76, 2.97)	1.59 (1.21, 2.10)	0.001
NAFLD or liver cirrhosis	2640	178/2098 (8.5)	40/542 (7.4)	0.86 (0.60, 1.23)	1.10 (0.76, 1.59)	0.63
Active cancer	2742	194/2175 (8.9)	59/567 (10.4)	1.19 (0.87, 1.61)	0.89 (0.65, 1.22)	0.47
COPD	2732	180/2168 (8.3)	83/564 (14.7)	1.91 (1.44, 2.52)	1.58 (1.18, 2.10)	0.002
Treated OSA	2593	211/2049 (10.3)	62/544 (11.4)	1.12 (0.83, 1.51)	1.22 (0.89, 1.67)	0.21
Routine treatment before admission						
Metformin	2794	1306/2217 (58.9)	247/577 (42.8)	0.52 (0.43, 0.63)	0.63 (0.52, 0.77)	<0.001
Sulfonylurea/glinides	2794	633/2217 (28.6)	149/577 (25.8)	0.87 (0.71, 1.07)	0.83 (0.67, 1.03)	0.093
DPP4-inhibitors	2794	502/2217 (22.6)	113/577 (19.6)	0.83 (0.66, 1.05)	0.83 (0.65, 1.05)	0.12
GLP-1RA	2794	221/2217 (10.0)	33/577 (5.7)	0.55 (0.38, 0.80)	0.78 (0.53, 1.15)	0.21
Insulin	2796	762/2219 (34.3)	277/577 (48.0)	1.77 (1.47, 2.12)	1.72 (1.41, 2.08)	<0.001
Diuretics^d^	2794	768/2217 (34.6)	290/577 (50.3)	1.91 (1.58, 2.29)	1.48 (1.22, 1.80)	<0.001
β-Blockers	2794	732/2217 (33.0)	256/577 (44.4)	1.62 (1.34, 1.95)	1.27 (1.04, 1.54)	0.017
CCB	2794	710/2217 (32.0)	214/577 (37.1)	1.25 (1.03, 1.51)	1.17 (0.96, 1.43)	0.12
ARBs and/or ACE inhibitors and/or MRA^e^	2794	1219/2217 (55.0)	351/577 (60.8)	1.27 (1.05, 1.53)	1.15 (0.94, 1.40)	0.17
Statins	2794	983/2217 (44.3)	299/577 (51.8)	1.35 (1.12, 1.62)	1.27 (1.05, 1.54)	0.015
Anti-platelet therapy	2794	834/2217 (37.6)	291/577 (50.4)	1.69 (1.40, 2.03)	1.34 (1.11, 1.63)	0.003
Anticoagulation therapy	2794	352/2217 (15.9)	149/577 (25.8)	1.84 (1.48, 2.29)	1.25 (0.99, 1.58)	0.057

Population size was *N* = 2796. Data shown are *n* (%), and mean ± SD or median (25th–75th percentile) if not normally distributed

*p* values are calculated using Wald test (unadjusted and age-adjusted logistic regression, except for ‘age’ and ‘age class’, which were not adjusted). ORs correspond to an increase of 1 SD after natural-log transformation and standardisation for BMI, diabetes duration and HbA_1c_

^a^HbA_1c_ determined in the 6 months prior to or in the first 7 days following hospital admission

^b^Microvascular complication was defined as history of one or more of the following: diabetic kidney disease and/or severe diabetic retinopathy and/or diabetic foot ulcer

^c^Macrovascular complication was defined as history of one or more of the following comorbidities: ischaemic heart disease (acute coronary syndrome and/or coronary artery revascularisation) and/or cerebrovascular disease (stroke and/or transient ischaemic attack) and/or peripheral artery disease (amputation owing to ischaemic disease and/or lower limb artery revascularisation)

^d^Diuretics stands here for loop diuretics, thiazide diuretics, and potassium-sparing diuretics

^e^MRAs include spironolactone and eplerenone

Abbreviations: EU, Europid; MENA, Middle East North Africa; AC, African or Caribbean; AS, Asian; NAFLD, non-alcoholic fatty liver disease; GLP-1RA, glucagon-like peptide 1-receptor agonist

When considering the characteristics on admission, COVID-19 symptoms, fever and dyspnoea were positively associated with death within 28 days after adjustment for age (Table 4). Nearly all biological covariates reflecting COVID-19 severity were associated with death, including admission plasma glucose, plasma creatinine, AST, white cells, lymphocyte and platelet counts and CRP level.

**Table 4:** Characteristics on admission in CORONADO participants, according to death within 28 days

		Death within 28 days				
Variable	Available data	No (*n* = 2219)	Yes (*n* = 577)	Unadjusted OR (95% CI)^a^	Age-adjusted OR (95% CI)^a^	Age-adjusted *p* value
Time from symptom onset to hospital admission (days)	2743	6 (3–9)	4 (1–7)	0.94 (0.92, 0.96)	0.97 (0.95, 0.98)	<0.001
Clinical presentation						
Fever	2755	1647/2190 (75.2)	430/565 (76.1)	1.05 (0.85, 1.30)	1.28 (1.02, 1.60)	0.034
Dyspnoea	2754	1338/2188 (61.2)	433/566 (76.5)	2.07 (1.67, 2.56)	2.34 (1.87, 2.92)	<0.001
Abnormal chest CT scan	1982	1560/1615 (96.6)	359/367 (97.8)	1.58 (0.75, 3.35)	2.05 (0.95, 4.46)	0.069
Biological findings						
Positive SARS-CoV-2 PCR	2705	2018/2155 (93.6)	529/550 (96.2)	1.71 (1.07, 2.73)	1.92 (1.18, 3.12)	0.008
Admission plasma glucose (mmol/l)	1551	9.2 (7.0–13.2)	10.2 (7.1–14.5)	1.07 (0.94, 1.21)	1.21 (1.06, 1.38)	0.005
Plasma creatinine (μmol/l)	2602	87 (66–121)	121 (85–185)	1.69 (1.54, 1.85)	1.65 (1.49, 1.81)	<0.001
eGFR (ml min^−1^ [1.73 m]^−2^)^a^	2602	74.8 (47.6–93.1)	45.7 (27.2–70.1)	0.56 (0.51, 0.61)	0.62 (0.56, 0.68)	<0.001
ALT (%ULN)	2478	0.63 (0.43–1.00)	0.59 (0.39–0.98)	1.01 (0.92, 1.12)	1.15 (1.05, 1.27)	0.004
AST (%ULN)	2444	1.03 (0.72–1.50)	1.33 (0.87–2.03)	1.46 (1.33, 1.60)	1.58 (1.43, 1.75)	<0.001
GGT (%ULN)	2317	0.95 (0.56–1.75)	0.95 (0.54–1.83)	1.04 (0.94, 1.16)	1.10 (0.99, 1.22)	0.067
Haemoglobin (g/l)	2728	128 (115–142)	124 (109–139)	0.81 (0.74, 0.89)	0.86 (0.78, 0.95)	0.003
White cell count (10^3^/mm^3^)	2726	6450 (4900–8510)	7260 (5420–10,100)	1.35 (1.23, 1.48)	1.34 (1.22, 1.48)	<0.001
Lymphocyte count (10^3^/mm^3^)	2646	1015 (710–1430)	845 (550–1200)	0.74 (0.66, 0.82)	0.80 (0.72, 0.89)	<0.001
Platelet count (10^3^/mm^3^)	2725	205 (159–263)	186 (142–241)	0.77 (0.70, 0.84)	0.79 (0.72, 0.87)	<0.001
CRP (mg/l)	2612	80 (36–140)	111 (59–181)	1.49 (1.33, 1.66)	1.62 (1.44, 1.82)	<0.001
LDH (U/l)	1427	338 (257–464)	436 (311–620)	1.97 (1.61, 2.42)	2.20 (1.78, 2.72)	<0.001
CPK (U/l)	1385	120 (63–265)	202 (82–485)	1.45 (1.28, 1.65)	1.52 (1.33, 1.74)	<0.001
Fibrinogen (g/l)	1424	6.3 (5.0–7.4)	6.3 (4.9–7.5)	1.04 (0.92, 1.18)	1.10 (0.97, 1.24)	0.13

Population size was *N* = 2796. Data shown are *n* (%) and or median (25th–75th percentile)

*p* values are calculated using Wald test (unadjusted and age-adjusted logistic regression). Quantitative variables were natural-log transformed and associated ORs correspond to an increase of 1 SD after standardisation, except for time from symptoms onset to hospital admission (1 day increase)

^a^eGFR determined by the CKD-EPI formula

Abbreviations: ALT, alanine aminotransferase; ULN, upper limit of normal; GGT, γ-glutamyl transferase; LDH, lactate dehydrogenase; CPK, creatine phosphokinase

Multivariable models for death within 28 days are presented in Fig. 2. After multiple adjustment, older age, history of microvascular complications, insulin and statin routine medication, dyspnoea on admission, higher AST, higher white cell count, lower platelet count and higher CRP level were associated with a greater risk of death when routine metformin therapy and a longer time between symptom onset and hospital admission were negatively associated with death. In a sensitivity analysis restricted to participants with positive SARS-CoV-2 PCR or those with available admission plasma glucose, the results were similar (ESM Table 8).

### Outcomes within 7 days

In the first 7 days following hospital admission, the prespecified primary outcome (tracheal intubation for mechanical ventilation and/or death) occurred in 800 (28.6%) participants and death in 312 (11.2%). The phenotypic characteristics of CORONADO participants according to the primary outcome are described in ESM Table 2 (features prior to admission) and ESM Table 3 (features on admission). Multivariable analysis (*n* = 1335, ESM Table 4) identified dyspnoea on admission, higher AST, higher white cell count, lower platelet count and higher CRP level as positively associated and metformin use as negatively associated with the primary outcome. The same results were observed in a sensitivity analysis on the complete-case population restricted to patients with positive PCR (*n* = 1249) or with available plasma glucose on admission (*n* = 761) (ESM Table 4).

## Discussion

CORONADO is the first registered study that specifically aimed to describe the phenotypic characteristics and to identify prognostic factors in patients with diabetes hospitalised for COVID-19. The present analysis reports the results of the complete CORONADO study totalling 2796 participants from 68 centres with complete follow-up up to day 28. We noted that one out of two CORONADO participants returned home by day 28. Younger age, routine metformin therapy and a longer duration of symptoms before admission were independently associated with such a favourable outcome, while a history of microvascular complications, routine anticoagulant therapy, dyspnoea and biological markers of COVID-19 severity were associated with a reduced chance of discharge by day 28. In contrast, BMI, HbA_1c_ and other comorbidities were not associated with this outcome. Results for death within 28 days mirrored the factors found for hospital discharge in an opposite way.

Regarding death, we reported a mortality rate of 11.2% within 7 days which reached 20.6% within 28 days. These findings can be compared to international reports. In a meta-analysis considering a majority of Chinese studies, in-hospital mortality was encountered in 299/2571 (11.6%) but the severity of illness in those patients is questionable since they were admitted early in the pandemic [15]. In contrast, in a retrospective cohort analysis of 1126 patients with diabetes hospitalised with COVID-19 at a large academic medical centre in New York City, the mortality rate was 33.1%, much higher than our findings. The higher prevalence of cardiovascular and chronic kidney disease, both independently associated with mortality, could explain this discrepancy [16]. In the UK, Docherty et al reported data on 20,133 hospitalised COVID-19 patients including 4949 with diabetes (1299 with and 3650 without diabetes complications) [17]. This study is consistent with our findings, with a population of similar age (mean age: 73 years in the UK study vs 70 years in CORONADO). In-hospital death occurred in 1080/3650 patients with uncomplicated diabetes (29.6%) and in 389/1299 (29.9%) with complicated diabetes, although the observational period is not directly mentioned. We therefore believe that our data are generalisable, at least to people living with diabetes in Europe.

With regard to prognostic factors of death, our results are in accordance with three nationwide studies from the UK. Holman et al evidenced that death was associated with age and male sex, but also with deprivation, which is not available in our report, while a U-shaped relationship was found for HbA_1c_ [18]. OpenSAFELY was able to examine, through electronic health record analysis, a population of more than 17 million people linked to more than 10,000 COVID-related deaths and showed that death was associated with male sex, age, BMI, and a clearly graded effect of HbA_1c_: the higher the HbA_1c_ the greater the risk of death [19], while we could not show this for HbA_1c_ in our more limited dataset. More recently Knight et al computed a prognostic score of in-hospital death considering a population with over 20% of people affected by diabetes. In accordance with our findings, they also identified age, sex and CRP as prognostic factors while other factors were not directly available in our study [20].

However, these papers did not mention hospital discharge and consider general populations and mostly ambulatory patients, which is at variance with what was performed in the CORONADO study.

It should be noted that the numbers of patients who died during follow-up were close to being equally distributed within the first 7 days (11.2%) and day 8 to day 28 (9.5%), while tracheal intubation for mechanical ventilation occurred, as expected, predominantly within 7 days after admission (532/556, 95.6%). These findings are in line with the previously described clinical course of the disease. Thus, in 54 non-survivors of COVID-19 from Wuhan, China, the median hospital length of stay was of 7.5 days [2]. Moreover, Argenziano et al found that 71.6% of 225 patients were intubated within the first 3 days following admission to the emergency room [7].

In addition to data on in-hospital death, we believe data on home discharge within 28 days should be reported since it is a positive result of value for both patients and physicians and it provides another perspective on COVID-19. Our data support the reasonable opinion that factors associated with home discharge mirror, as expected, those associated with the severity of the disease. This turned out to be true with biological covariates on admission such as plasma creatinine, AST and CRP, with a particular importance for admission blood glucose.

While our multivariable analyses were performed on a limited number of participants, owing to missing data, the current study helped to establish the relative contribution of admission blood glucose on the risk of death and on the chance of discharge. We found that chronic glycaemic control assessed with pre-admission or admission HbA_1c_ did not impact on the fate of COVID-19 patients, with no significant association with death or with discharge within 28 days. In contrast, increased admission plasma glucose concentration was a strong predictor of death and, consistently, of a lower chance of discharge. We have recently commented on two interesting studies reporting on the question of admission plasma glucose in COVID-19 and community-acquired pneumonia [21, 22]. This association between blood glucose control and prognosis in hospitalised COVID-19 patients has also been observed in patients without previously diagnosed diabetes [23–25]. Furthermore, other studies suggest that blood glucose control during hospitalisation is associated with COVID-19 outcomes [26, 27]. However, due to the observational design of our study and others, no conclusion regarding causality can be drawn. The relationship between admission plasma glucose and severe conditions was previously established in many clinical situations, including, for instance, heart failure, stroke or myocardial infarction [28–31]. In none of these situations did strict metabolic control consistently decrease mortality.

Our results also establish an association between routine therapy and chance of discharge or risk of death. Interestingly, metformin was associated with favourable outcomes (both lower risk of death and greater chance of discharge within 28 days). Conversely, statin use was associated with higher risk of death and anticoagulation therapy with a reduced chance of discharge. The findings with statins are surprising since the in-hospital use of this drug class has been recently associated with a lower risk of 28-day mortality in 13,981 patients with COVID-19 from China [32]. Unexpectedly, we also found a higher risk of primary outcomes within 7 days (but not of other outcomes) in patients taking calcium channel blockers (CCBs) on admission. However, whether these findings reflect a significant negative effect or a mere indication bias would require more sophisticated adjustment methods such as specific propensity score, which is beyond the scope of the current paper.

### Study limitations

Some limitations must be acknowledged here. First, we focused on hospitalised COVID-19 cases and our results cannot be generalised to all COVID-19 patients with diabetes, especially those with a less severe form of the disease. Second, we focused on those discharged to their home or to a long-term care facility. Since insufficient ascertainment was available from those transferred to another hospital or rehabilitation centre, our figures are conservative even if we believe that transfer is unlikely to lead to short-term discharge before day 28. Third, it can also be argued that the 28-day observation period was too short with 12.2% of admitted participants requiring more prolonged hospitalisation in the same hospital. Nonetheless, this 28-day mark is a well-established milestone, particularly in an ICU setting, and further outcomes probably reflect other medical issues. Fourth, for a non-negligible number of patients (249/2796, 8.9%), SARS-CoV-2 PCR was not available or negative. However, the main analyses were replicated in the subset with a positive SARS-CoV-2 PCR showing similar results. Fifth, due to the exploratory nature of the results, our work must be read as a post hoc analysis. The chosen multivariable approach infers, by design, obvious limitations: a lack of control of type 1 error; the transformation of quantitative variables, which was needed to comply with the statistical assumption of the model, at the expense of less natural clinical interpretation; and the adjustment set, based on background knowledge, which is associated with a risk of overfitting. Sixth, although specific treatment for COVID-19, such as corticosteroid, immunotherapy or antiviral agents could influence prognosis, data about such treatment given during hospitalisation were not available in our study. Ultimately, our results are hampered by missing data, such as HbA_1c_. Nevertheless, the CORONADO study is, to date, the most accurate study providing such information. The inclusion of additional studies of a similar nature would help to increase our confidence in observations with significant missing data.

The study strengths must also be acknowledged, such as the originality of the medical question leading to the CORONADO initiative and the inclusion of participants with a nationwide coverage, in any department of a participating hospital, including the ICU and medical units. We also recorded patients’ phenotypic characteristics in a structured manner and did not solely rely on medico-administrative databases.

In conclusion, the CORONADO study refined the phenotypes of hospitalised COVID-19 patients with diabetes. To summarise, younger age, routine metformin therapy and longer symptom duration on admission were positively associated with discharge. History of microvascular complications, anticoagulant routine therapy, dyspnoea on admission, and higher AST, white cell count and CRP levels were associated with a reduced chance of discharge. Deleterious and protective factors for discharge mirrored those for death by day 28, respectively. The identification of favourable variables associated with hospital discharge and deleterious variables associated with death can lead to patient reclassification and help to use resources adequately according to individual patient profile.

## Supplementary Information

ESM 1 (PDF 1.00 mb)

## Data Availability

No sharing of participant data is allowed by our regulatory authorities. So far, French regulations have not validated deidentified data or avatars for data sharing. Our statement might be modified in the case that French law changes.

## Abbreviations

ARB: Angiotensin-2 receptor blockers
AST: Aspartate aminotransferase
CCB: Calcium channel blocker
COPD: Chronic obstructive pulmonary disease
CORONADO: Coronavirus-SARS-CoV-2 and Diabetes Outcomes
COVID-19: Coronavirus disease-2019
CRP: C-reactive protein
CT: Computed tomography
DPP-4: Dipeptidyl peptidase 4
ICU: Intensive care unit
MRA: Mineralocorticoid receptor antagonist
OSA: Obstructive sleep apnoea
SARS-CoV-2: Severe acute respiratory syndrome coronavirus-2

## References

[1] Onder G, Rezza G, Brusaferro S (2020). Case-fatality rate and characteristics of patients dying in relation to COVID-19 in Italy. JAMA..

[2] Zhou F, Yu T, Du R (2020). Clinical course and risk factors for mortality of adult inpatients with COVID-19 in Wuhan, China: a retrospective cohort study. Lancet.

[3] Alqahtani FY, Aleanizy FS, Ali El Hadi Mohamed R et al (2018) Prevalence of comorbidities in cases of Middle East respiratory syndrome coronavirus: a retrospective study. Epidemiol Infect:1–5. 10.1017/S0950268818002923 10.1017/S0950268818002923 PMC6518603 30394248

[4] Yang JK, Feng Y, Yuan MY (2006). Plasma glucose levels and diabetes are independent predictors for mortality and morbidity in patients with SARS. Diabet Med.

[5] Grasselli G, Zangrillo A, Zanella A (2020). Baseline characteristics and outcomes of 1591 patients infected with SARS-CoV-2 admitted to ICUs of the Lombardy region, Italy. JAMA.

[6] Selvin E, Juraschek SP (2020). Diabetes epidemiology in the COVID-19 pandemic. Diabetes Care.

[7] Argenziano MG, Bruce SL, Slater CL (2020). Characterization and clinical course of 1000 patients with coronavirus disease 2019 in New York: retrospective case series. BMJ.

[8] Bhatraju PK, Ghassemieh BJ, Nichols M (2020). Covid-19 in critically ill patients in the Seattle region - case series. N Engl J Med.

[9] Roncon L, Zuin M, Rigatelli G, Zuliani G (2020). Diabetic patients with COVID-19 infection are at higher risk of ICU admission and poor short-term outcome. J Clin Virol.

[10] Wu C, Chen X, Cai Y (2020). Risk factors associated with acute respiratory distress syndrome and death in patients with coronavirus disease 2019 pneumonia in Wuhan, China. JAMA Intern Med.

[11] Joensen LE, Madsen KP, Holm L (2020). Diabetes and COVID-19: psychosocial consequences of the COVID-19 pandemic in people with diabetes in Denmark-what characterizes people with high levels of COVID-19-related worries?. Diabet Med.

[12] Mukhtar S, Mukhtar S (2020). Letter to the editor: mental health and psychological distress in people with diabetes during COVID-19. Metabolism.

[13] Sy SL, Munshi MN (2020). Caring for older adults with diabetes during the COVID-19 pandemic. JAMA Intern Med.

[14] Cariou B, Hadjadj S, Wargny M (2020). Phenotypic characteristics and prognosis of inpatients with COVID-19 and diabetes: the CORONADO study. Diabetologia.

[15] Mantovani A, Byrne CD, Zheng MH, Targher G (2020). Diabetes as a risk factor for greater COVID-19 severity and in-hospital death: a meta-analysis of observational studies. Nutr Metab Cardiovasc Dis.

[16] Agarwal S, Schechter C, Southern W, Crandall JP, Tomer Y (2020). Preadmission diabetes-specific risk factors for mortality in hospitalized patients with diabetes and coronavirus disease 2019. Diabetes Care.

[17] Docherty AB, Harrison EM, Green CA (2020). Features of 20 133 UK patients in hospital with covid-19 using the ISARIC WHO Clinical Characterisation Protocol: prospective observational cohort study. BMJ.

[18] Holman N, Knighton P, Kar P (2020). Risk factors for COVID-19-related mortality in people with type 1 and type 2 diabetes in England: a population-based cohort study. Lancet Diabetes Endocrinol.

[19] Williamson E, Walker A, Bhaskaran K (2020). Factors associated with COVID-19-related death using OpenSAFELY. Nature.

[20] Knight S, Ho A, Pius R (2020). Risk stratification of patients admitted to hospital with covid-19 using the ISARIC WHO Clinical Characterisation Protocol: development and validation of the 4C Mortality Score. BMJ.

[21] Sardu C, D’Onofrio N, Balestrieri ML (2020). Hyperglycaemia on admission to hospital and COVID-19. Diabetologia.

[22] Lepper PM, Bals R, Jüni P, von Eynatten M (2020). Blood glucose, diabetes and metabolic control in patients with community-acquired pneumonia. Diabetologia.

[23] Wang S, Ma P, Zhang S (2020). Fasting blood glucose at admission is an independent predictor for 28-day mortality in patients with COVID-19 without previous diagnosis of diabetes: a multi-centre retrospective study. Diabetologia.

[24] Zhang B, Liu S, Zhang L, Dong Y, Zhang S (2020) Admission fasting blood glucose predicts 30-day poor outcome in patients hospitalized for COVID-19 pneumonia. Diabetes Obes Metab. 10.1111/dom.14132 10.1111/dom.14132 PMC7361510 32627338

[25] Coppelli A, Giannarelli R, Aragona M (2020). Hyperglycemia at hospital admission is associated with severity of the prognosis in patients hospitalized for COVID-19: the Pisa COVID-19 Study. Diabetes Care.

[26] Xu Z, Wang Z, Wang S et al (2020) The impact of type 2 diabetes and its management on the prognosis of patients with severe COVID-19. J Diabetes. 10.1111/1753-0407.13084 10.1111/1753-0407.13084 PMC7361557 32638507

[27] Zhu L, She ZG, Cheng X (2020). Association of blood glucose control and outcomes in patients with COVID-19 and pre-existing type 2 diabetes. Cell Metab.

[28] Capes SE, Hunt D, Malmberg K, Gerstein HC (2000). Stress hyperglycaemia and increased risk of death after myocardial infarction in patients with and without diabetes: a systematic overview. Lancet.

[29] Gencer B, Rigamonti F, Nanchen D et al (2018) Prognostic values of fasting hyperglycaemia in non-diabetic patients with acute coronary syndrome: a prospective cohort study. Eur Heart J Acute Cardiovasc Care:2048872618777819. 10.1177/2048872618777819 10.1177/2048872618777819 29862825

[30] Itzhaki Ben Zadok O, Kornowski R, Goldenberg I (2017). Admission blood glucose and 10-year mortality among patients with or without pre-existing diabetes mellitus hospitalized with heart failure. Cardiovasc Diabetol.

[31] Pan Y, Cai X, Jing J (2017). Stress hyperglycemia and prognosis of minor ischemic stroke and transient ischemic attack: the CHANCE study (clopidogrel in high-risk patients with acute nondisabling cerebrovascular events). Stroke.

[32] Zhang XJ, Qin JJ, Cheng X et al (2020) In-hospital use of statins is associated with a reduced risk of mortality among individuals with COVID-19. Cell Metab 32(2):176–187.e4. 10.1016/j.cmet.2020.06.015 10.1016/j.cmet.2020.06.015 PMC7311917 32592657

