# Supplementary material for: Predictors of hospital discharge and mortality in patients with diabetes and COVID-19: updated results from the nationwide CORONADO study
Source: Diabetologia. 2021 Feb 17;64(4):778–94. doi: 10.1007/s00125-020-05351-w (PMC7890396; doi:10.1007/s00125-020-05351-w)
Supplement: Supplementary file 1 — (PDF 1.00 mb) [file 125_2020_5351_MOESM1_ESM.pdf]

**Wargny M, Potier L, Gourdy P et al.**

# Predictors of Hospital Discharge and Mortality in Patients with Diabetes and Covid-19: Updated Results from the Nationwide CORONADO Study.

## ESM TABLES

**ESM Table 1** Upper limit of normal for liver enzymes (AST, ALT, GGT) according to centre and patients' sex.

**ESM Table 2** Clinical characteristics prior to admission of CORONADO participants, according to the composite outcome within 7 days

**ESM Table 3** Characteristics on admission in CORONADO participants, according to the composite outcome within 7 days

**ESM Table 4** Multivariable analysis of the composite outcome within 7 days, complete case population and restricted to CORONADO participants with positive PCR or available admission plasma glucose: covariates prior to and on admission

**ESM Table 5** COVID-19-related clinical outcomes within the first 7 and 28 days following hospital admission.

**ESM Table 6** Multivariable analysis of discharge and death within 28 days in complete case population, with raw features before transformation

**ESM Table 7** Multivariable analysis of discharge within 28 days, complete case population and restricted to CORONADO participants with positive PCR or available admission plasma glucose: covariates prior to and on admission

**ESM Table 8** Multivariable analysis of death within 28 days, complete case population and restricted to CORONADO participants with positive PCR or available admission plasma glucose: covariates prior to and on admission

## ESM FIGURES

**ESM Fig. 1** Flow diagram

**ESM Fig. 2a,b** Length of stay before discharge according to admission date, in individuals who returned home within 28 days (N = 1404).

## ESM TEXT

**ESM Text 1** List of the collaborators of the CORONADO study

## ESM TABLES

### ESM Table 1 Upper limit of normal for liver enzymes (AST, ALT, GGT) according to centre and patients' sex

| | | AST, upper limit of normal (U/l) | | ALT, upper limit of normal (U/l) | | GGT – upper limit of normal (U/l) | |
|---|---|---|---|---|---|---|---|
| N° | Centres (opening order) | Women | Men | Women | Men | Women | Men |
| 1 | CHU Nantes | 36 | 51 | 36 | 51 | 42 | 71 |
| 2 | CHU Toulouse - Rangueil | 35 | 50 | 28 | 40 | 40 | 60 |
| 3 | AP-HP - Lariboisière, Paris | 34 | 34 | 55 | 55 | 36 | 64 |
| 4 | AP-HP - Bichat, Paris | 37 | 37 | 59 | 63 | 55 | 85 |
| 5 | HCL Sud, Lyon | 34 | 34 | 55 | 55 | 36 | 36 |
| 6 | Hôpitaux Universitaires de Strasbourg - Hôpital Civil | 34 | 34 | 41 | 59 | 40 | 68 |
| 7 | CHU Nancy | 40 | 40 | 40 | 40 | 38 | 73 |
| 8 | CHRU Lille | 35 | 50 | 35 | 50 | 30 | 50 |
| 9 | CHU Bordeaux | 34 | 34 | 55 | 55 | 36 | 64 |
| 10 | CHU Poitiers | 50 | 50 | 50 | 50 | 36 | 61 |
| 11 | CHU Montpellier | 32 | 40 | 33 | 41 | 40 | 60 |
| 12 | CHU de Brest | 34 | 34 | 49 | 49 | 38 | 73 |
| 13 | CHU Rennes - Pontchaillou | 31 | 35 | 34 | 45 | 38 | 55 |
| 14 | CHU Grenoble | 40 | 40 | 40 | 40 | 38 | 73 |
| 15 | AP-HP Avicenne-Bondy, Paris | 40 | 40 | 40 | 40 | 60 | 60 |
| 16 | CHU Caen | 31 | 35 | 34 | 45 | 38 | 55 |
| 17 | CHU Rouen | 35 | 50 | 35 | 50 | 38 | 55 |
| 18 | AP-HM - La Conception, Marseille | 35 | 50 | 35 | 50 | 40 | 60 |
| 19 | CHU Dijon | 37 | 37 | 56 | 61 | 55 | 85 |
| 20 | CHU Angers | 34 | 34 | 49 | 49 | 38 | 73 |
| 21 | CHU Nice | 50 | 50 | 50 | 50 | 61 | 61 |
| 22 | CHU Reims | 35 | 50 | 35 | 50 | 36 | 61 |
| 23 | Centre Hospitalier Sud Francilien, Corbeille-Essonnes | 32 | 40 | 33 | 41 | 40 | 60 |
| 24 | Hôpital d'Instruction des armées – Begin, Saint-Mandé | 32 | 40 | 33 | 41 | 40 | 60 |
| 25 | Groupe hospitalier Paris St Joseph, Paris | 31 | 35 | 34 | 45 | 36 | 64 |
| 26 | CH Cahors | 36 | 59 | 35 | 50 | 43 | 73 |
| 27 | CH Cayenne | 32 | 40 | 33 | 41 | 40 | 60 |
| 28 | Clinique Ambroise Paré, Neuilly-sur-Seine | 34 | 34 | 55 | 55 | 36 | 64 |
| 29 | CH Cholet | 32 | 40 | 33 | 41 | 40 | 60 |
| 30 | CHU Amiens | 40 | 40 | 40 | 40 | 38 | 73 |
| 31 | CHU Saint-Etienne | 50 | 50 | 50 | 50 | 60 | 60 |
| 32 | Hôpital Nord Franche Comté, Trévenans | 40 | 40 | 40 | 49 | 38 | 73 |
| 33 | CH Alès | 32 | 41 | 33 | 41 | 36 | 61 |
| 35 | CH Pontoise | 34 | 34 | 55 | 55 | 36 | 64 |
| 36 | CHU Ile de la Réunion | 32 | 40 | 55 | 55 | 36 | 64 |
| 37 | CH Libourne | 32 | 50 | 35 | 50 | 40 | 60 |
| 38 | CH Albi | 35 | 50 | 35 | 50 | 60 | 60 |
| 39 | CHU Chartres | 40 | 40 | 40 | 40 | 38 | 73 |
| 40 | CH Auch | | 40 | | 41 | | 60 |
| 41 | AP-HP Cochin, Paris | 45 | 45 | 35 | 45 | 35 | 55 |
| 42 | Hôpital Franco-Britannique Levallois-Perret | 34 | 34 | 55 | 55 | 78 | 78 |
| 43 | CH Forez | 50 | 50 | 50 | 50 | 60 | 60 |
| 44 | CHU Besançon | 34 | 34 | 55 | 55 | 36 | 64 |
| 46 | AP-HP Clamart le Kremlin-Bicêtre | 35 | 50 | 35 | 50 | 36 | 61 |
| 47 | CH Bretagne Atlantique, Vannes | 32 | 40 | 35 | 50 | 36 | 61 |
| 48 | CH Saint-Louis, La Rochelle | 34 | 34 | 55 | 55 | 64 | 64 |
| 49 | CH St Joseph St Luc, Lyon | 35 | 50 | 35 | 50 | 40 | 60 |
| 50 | CHD La Roche-sur-Yon | 35 | 50 | 35 | 50 | 36 | 61 |
| 51 | AP-HP La Pitié Salpêtrière, Paris | 27 | 32 | 26 | 35 | 36 | 55 |
| 55 | HCL-Sud, Lyon (nutrition) | 34 | 34 | 55 | 55 | 36 | 64 |
| 56 | HCL Bron, Lyon | 34 | 34 | 55 | 55 | 36 | 64 |
| 57 | AP-HP Saint Antoine, Paris | 32 | 35 | 32 | 43 | 32 | 45 |
| 58 | CH Côte Basque | 36 | 59 | 35 | 50 | 43 | 73 |
| 59 | CH Avignon | 35 | 50 | 34 | 45 | 42 | 71 |

**ESM Table 1 Upper limit of normal for liver enzymes (AST, ALT, GGT) according to centre and patients' sex (continued)**

| | | AST, upper limit of normal (U/L) | | ALT, upper limit of normal (U/L) | | GGT – upper limit of normal (U/L) | |
|---|---|---|---|---|---|---|---|
| N° | Centres (opening order) | Women | Men | Women | Men | Women | Men |
| 60 | CHU Fort-de-France, Ile de la Martinique | 34 | 34 | 55 | 55 | 36 | 64 |
| 61 | CH Aix | 34 | 34 | 55 | 55 | 33 | 59 |
| 63 | Hôpital Jacques Monod Le Havre | 35 | 50 | 35 | 50 | 42 | 71 |
| 64 | CH Agen | 34 | 34 | 55 | 55 | 36 | 64 |
| 66 | CH Le Mans | 35 | 50 | 35 | 50 | 40 | 60 |
| 67 | CH Gonesse | 37 | 37 | 61 | 61 | 85 | 85 |
| 68 | CHU Nîmes | 35 | 50 | 35 | 50 | 71 | 71 |
| 69 | CHU Guadeloupe | 32 | 40 | 34 | 42 | 42 | 71 |
| 71 | Hôpital St Vincent de Paul, Lille | 37 | 37 | 56 | 61 | 50 | 50 |
| 72 | CH Périgueux | 36 | 59 | 33 | 45 | 43 | 73 |
| 73 | CH Bastia | N/A | 34 | N/A | 55 | N/A | 64 |
| 74 | Groupe Hospitalier Mutualiste Les Portes du Sud, Vénissieux | 35 | 50 | 35 | 50 | 40 | 60 |
| 75 | Hôpital de Villeurbanne | 34 | 34 | 55 | 55 | 36 | 64 |
| 76 | CH Ajaccio | 35 | 50 | 33 | 50 | 36 | 64 |

Eight centres were candidates for the CORONADO study but did not include patients, which explain that 76 centres were selected but only 68 were recruiters and are presented here.

An empty box indicates that no patient of this sex was recruited in the centre, making the information irrelevant.

AST, aspartate aminotransferase; ALT, alanine aminotransferase; GGT, gamma-glutamyl transferase; AP-HM: *Assistance Publique Hôpitaux de Marseille* (Marseille's Public Hospitals); AP-HP: *Assistance Publique Hôpitaux de Paris* (Paris' Public Hospitals); CH: *Centre Hospitalier* (Hospital Centre); CHU: *Centre Hospitalier Universitaire (*University Hospital Centre); HCL: *Hospices Civils de Lyon* (Lyon's Public Hospitals); N/A: not available, applied when the centre did not communicate the upper limit of normal

**ESM Table 6 Clinical characteristics prior to admission of CORONADO participants according to the composite outcome (tracheal intubation for mechanical ventilation and/or death) within 7 days**

| | | Composite outcome within 7 days | | | | |
|---|---|---|---|---|---|---|
| **Clinical features** | **Available data** | **All (N = 2796)** | **No (N=1996)** | **Yes (N=800)** | **Age-adjusted OR (95% CI)** | **Age-adjusted *p* value** |
| **Sex (female/male)** | 2796 | 1014/2796 (36.3) | 776/1996 (38.9) | 238/800 (29.8) | 0.67 (0.56, 0.79) | <0.001 |
| **Age (years)** | 2796 | 69.7 ± 13.2 | 69.7 ± 13.4 | 69.6 ± 12.5 | 1.00 (0.99, 1.01) | 0.86 |
| **Age class (years)** | 2796 | | | | | 0.20 |
| < 55 | | 367/2796 (13.1) | 263/1996 (13.2) | 104/800 (13.0) | 1 | |
| 55-64 | | 565/2796 (20.2) | 410/1996 (20.5) | 155/800 (19.4) | 0.96 (0.71, 1.28) | 0.76 |
| 65-74 | | 797/2796 (28.5) | 546/1996 (27.4) | 251/800 (31.4) | 1.16 (0.89, 1.53) | 0.28 |
| ≥75 | | 1067/2796 (38.2) | 777/1996 (38.9) | 290/800 (36.2) | 0.94 (0.72, 1.23) | 0.67 |
| **Ethnicity** | 2384 | | | | | 0.19 |
| EU | | 1385/2384 (58.1) | 996/1715 (58.1) | 389/669 (58.1) | 1 | |
| MENA | | 497/2384 (20.8) | 366/1715 (21.3) | 131/669 (19.6) | 0.92 (0.73, 1.17) | 0.50 |
| AC | | 415/2384 (17.4) | 299/1715 (17.4) | 116/669 (17.3) | 1.01 (0.78, 1.31) | 0.94 |
| AS | | 87/2384 (3.6) | 54/1715 (3.1) | 33/669 (4.9) | 1.59 (1.01, 2.50) | 0.046 |
| **BMI (kg/m$^2$)** | 2460 | 28.4 (25; 32.4) | 28.1 (24.8; 32.1) | 29.1 (26; 33.5) | 1.21 (1.10, 1.32) | <0.001 |
| **BMI class** | 2460 | | | | | 0.005 |
| < 25 kg/m$^2$ | | 611/2460 (24.8) | 471/1759 (26.8) | 140/701 (20) | 1 | |
| 25-29.9 kg/m$^2$ | | 890/2460 (36.2) | 633/1759 (36) | 257/701 (36.7) | 1.35 (1.07, 1.72) | 0.013 |
| 30-39.9 kg/m$^2$ | | 823/2460 (33.5) | 562/1759 (31.9) | 261/701 (37.2) | 1.53 (1.20, 1.95) | 0.001 |
| ≥40 kg/m$^2$ | | 136/2460 (5.5) | 93/1759 (5.3) | 43/701 (6.1) | 1.51 (1.00, 2.27) | 0.051 |
| **Diabetes duration (years)** | 1767 | 11 (5; 20) | 11 (5; 20) | 11 (4; 20) | 0.96 (0.86, 1.07) | 0.49 |
| **HbA$_{1C}$ (mmol/mol)** [a] | 1800 | 60.7 (50.8; 74.9) | 60.7 (50.8; 74.9) | 59.6 (50.8; 73.2) | 0.94 (0.84, 1.05) | 0.28 |
| **HbA$_{1C}$ (%)** [a] | 1800 | 7.7 (6.8; 9.0) | 7.7 (6.8; 9.0) | 7.6 (6.8; 8.9) | 0.94 (0.84, 1.05) | 0.28 |
| **Hypertension** | 2769 | 2126/2769 (76.8) | 1494/1976 (75.6) | 632/793 (79.7) | 1.29 (1.05, 1.59) | 0.015 |
| **Dyslipidemia** | 2710 | 1267/2710 (46.8) | 890/1941 (45.9) | 377/769 (49) | 1.14 (0.96, 1.35) | 0.13 |
| **Current tobacco use** | 2288 | 131/2288 (5.7) | 96/1659 (5.8) | 35/629 (5.6) | 0.95 (0.64, 1.42) | 0.82 |
| **Long-term diabetes complications** | | | | | | |
| Microvascular complications[b] | 1966 | 869/1966 (44.2) | 626/1457 (43) | 243/509 (47.7) | 1.22 (0.99, 1.52) | 0.068 |
| Macrovascular complications[c] | 2627 | 1014/2627 (38.6) | 721/1889 (38.2) | 293/738 (39.7) | 1.07 (0.90, 1.29) | 0.44 |

**ESM Table 2 Clinical characteristics prior to admission of CORONADO participants according to the composite outcome (tracheal intubation for mechanical ventilation and/or death) within 7 days (continued)**

| | | Primary outcome within 7 days | | | | |
|---|---|---|---|---|---|---|
| **Clinical features** | **Available data** | **All (N = 2796)** | **No (N=1996)** | **Yes (N=800)** | **Age-adjusted OR (95% CI)** | **Age-adjusted *p* value** |
| **Comorbidities** | | | | | | |
| Heart failure | 2651 | 302/2651 (11.4) | 223/1904 (11.7) | 79/747 (10.6) | 0.89 (0.67, 1.17) | 0.41 |
| NAFLD or liver cirrhosis | 2640 | 218/2640 (8.3) | 153/1897 (8.1) | 65/743 (8.7) | 1.09 (0.81, 1.48) | 0.57 |
| Active Cancer | 2742 | 253/2742 (9.2) | 184/1962 (9.4) | 69/780 (8.8) | 0.94 (0.70, 1.26) | 0.68 |
| COPD | 2732 | 263/2732 (9.6) | 183/1956 (9.4) | 80/776 (10.3) | 1.12 (0.85, 1.48) | 0.42 |
| Treated OSA | 2593 | 273/2593 (10.5) | 180/1864 (9.7) | 93/729 (12.8) | 1.37 (1.05, 1.79) | 0.021 |
| **Routine treatment before admission** | | | | | | |
| Metformin | 2794 | 1553/2794 (55.6) | 1119/1994 (56.1) | 434/800 (54.2) | 0.92 (0.78, 1.09) | 0.35 |
| Sulfonylurea/glinides | 2794 | 782/2794 (28.0) | 547/1994 (27.4) | 235/800 (29.4) | 1.10 (0.92, 1.32) | 0.30 |
| DPP4-inhibitors | 2794 | 615/2794 (22.0) | 446/1994 (22.4) | 169/800 (21.1) | 0.93 (0.76, 1.14) | 0.48 |
| GLP1-RA | 2794 | 254/2794 (9.1) | 178/1994 (8.9) | 76/800 (9.5) | 1.07 (0.80, 1.42) | 0.65 |
| Insulin | 2796 | 1039/2796 (37.2) | 754/1996 (37.8) | 285/800 (35.6) | 0.91 (0.77, 1.08) | 0.29 |
| Diuretics [d] | 2794 | 1058/2794 (37.9) | 745/1994 (37.4) | 313/800 (39.1) | 1.09 (0.91, 1.29) | 0.35 |
| β-Blockers | 2794 | 988/2794 (35.4) | 697/1994 (35.0) | 291/800 (36.4) | 1.07 (0.90, 1.27) | 0.45 |
| CCB | 2794 | 924/2794 (33.1) | 619/1994 (31.0) | 305/800 (38.1) | 1.37 (1.16, 1.63) | <0.001 |
| ARBs and/or ACE inhibitors and/or MRA [e] | 2794 | 1570/2794 (56.2) | 1108/1994 (55.6) | 462/800 (57.8) | 1.10 (0.93, 1.30) | 0.28 |
| Statins | 2794 | 1282/2794 (45.9) | 899/1994 (45.1) | 383/800 (47.9) | 1.12 (0.95, 1.32) | 0.17 |
| Anti-platelet therapy | 2794 | 1125/2794 (40.3) | 793/1994 (39.8) | 332/800 (41.5) | 1.08 (0.91, 1.28) | 0.37 |
| Anticoagulation therapy | 2794 | 501/2794 (17.9) | 382/1994 (19.2) | 119/800 (14.9) | 0.73 (0.58, 0.92) | 0.007 |

Population size was *N*=2796. Data shown are number (%), and mean ± SD or median (25th–75th percentile) if not normally distributed

*p* values are calculated using Wald test (unadjusted and age-adjusted logistic regression, except for 'age' and 'age class', which were not adjusted). ORs correspond to an increase of 1 SD after natural-log transformation and standardisation for BMI, diabetes duration and $HbA_{1c}$

[a]$HbA_{1c}$ determined in the 6 months prior to or in the first 7 days following hospital admission

[b] Microvascular complication was defined as history of one or more of the following: diabetic kidney disease and/or severe diabetic retinopathy and/or diabetic foot ulcer

[c]Macrovascular complication was defined as history of one or more of the following comorbidities: ischemic heart disease (acute coronary syndrome and/or coronary artery disease revascularisation) and/or cerebrovascular disease (stroke and/or transient ischaemic attack) and/or peripheral heart disease (amputation owing to ischemic disease and/or lower limb artery revascularisation)

[d]Diuretics stands here for loop diuretics, thiazide diuretics, and potassium-sparing diuretics

[e]MRAs include spironolactone and eplerenone

Abbreviations: EU, Europid; MENA, Middle East North Africa; AC, African or Caribbean; AS, Asian; NAFLD, non-alcoholic fatty liver disease; GLP-1RA, glucagon-like peptide 1-receptor agonist

**ESM Table 3 Characteristics on admission of CORONADO participants according to the composite outcome (tracheal intubation for mechanical ventilation and/or death) within 7 days**

| | | Composite outcome within 7 days | | | | |
|---|---|---|---|---|---|---|
| Variable | Available data | All (N = 2796) | No (N=1996) | Yes (N=800) | Age-adjusted OR (95% CI) | Age-adjusted *p* value |
| **Time from symptom onset to hospital admission (days)** | 2743 | 5 (2-9) | 5 (2-9) | 6 (3-8) | 0.99 (0.98, 1.01) | 0.42 |
| **Clinical presentation** | | | | | | |
| Fever | 2755 | 2077/2755 (75.4) | 1455/1976 (73.6) | 622/779 (79.8) | 1.42 (1.16, 1.75) | 0.001 |
| Dyspnoea | 2754 | 1771/2754 (64.3) | 1138/1972 (57.7) | 633/782 (80.9) | 3.12 (2.55, 3.80) | <0.001 |
| **Abnormal chest CT scan** | 1982 | 1919/1982 (96.8) | 1395/1449 (96.3) | 524/533 (98.3) | 2.29 (1.12, 4.67) | 0.023 |
| **Biological findings** | | | | | | |
| Positive SARS-CoV-2 PCR | 2705 | 2547/2705 (94.2) | 1795/1934 (92.8) | 752/771 (97.5) | 3.06 (1.88, 4.98) | <0.001 |
| Admission plasma glucose (mmol/l) | 1551 | 9.5 (7.0-13.5) | 9.0 (6.9-12.8) | 10.5 (7.5-14.9) | 1.25 (1.12, 1.39) | <0.001 |
| Plasma creatinine (µmol/l) | 2602 | 91 (69-133) | 88 (67-125) | 104 (76-155) | 1.33 (1.23, 1.45) | <0.001 |
| eGFR (ml $min^{-1}$ $[1.73\ m]^{-2}$) [a] | 2602 | 68.5 (41.8-89.7) | 72.4 (44.7-91.0) | 58.7 (34.8-86.0) | 0.77 (0.71, 0.84) | <0.001 |
| ALT (%ULN) | 2478 | 0.62 (0.42; 1.00) | 0.58 (0.40; 0.94) | 0.73 (0.48; 1.11) | 1.33 (1.22, 1.45) | <0.001 |
| AST (%ULN) | 2444 | 1.06 (0.75; 1.60) | 0.98 (0.69; 1.40) | 1.38 (0.94; 2.16) | 1.74 (1.58, 1.92) | <0.001 |
| GGT (%ULN) | 2317 | 0.95 (0.55; 1.77) | 0.91 (0.53; 1.62) | 1.10 (0.65; 2.00) | 1.21 (1.10, 1.31) | <0.001 |
| Hemoglobin (g/l) | 2728 | 127 (114-142) | 127 (114-142) | 128 (113-143) | 0.99 (0.91, 1.08) | 0.84 |
| White cell count ($10^3/mm^3$) | 2726 | 6580 (5000-8818) | 6410 (4900-8500) | 7050 (5380-9960) | 1.36 (1.25, 1.48) | <0.001 |
| Lymphocyte count ($10^3/mm^3$) | 2646 | 990 (690-1400) | 1040 (720-1470) | 860 (600-1200) | 0.72 (0.65, 0.80) | <0.001 |
| Platelet count ($10^3/mm^3$) | 2725 | 201 (156-260) | 206 (162-265) | 188 (145-241) | 0.81 (0.75, 0.88) | <0.001 |
| CRP (mg/l) | 2612 | 86.0 (40.3-148.0) | 71.4 (31.7-126.0) | 123.0 (72.7-195.9) | 2.22 (1.97, 2.50) | <0.001 |
| LDH (U/l) | 1427 | 351 (266-498) | 322 (248-428) | 456 (332-626) | 2.63 (2.17, 3.18) | <0.001 |
| CPK (U/l) | 1385 | 132 (67-305) | 112 (61-233) | 212 (90-506) | 1.63 (1.45, 1.83) | <0.001 |
| Fibrinogen (g/l) | 1424 | 6.3 (5.0-7.4) | 6.1 (4.8-7.2) | 6.6 (5.3-7.8) | 1.24 (1.10, 1.40) | 0.001 |

Population size was *N*=2796. Data shown are *n* (%) and or median (25th–75th percentile)

*p* values are calculated using Wald test (unadjusted and age-adjusted logistic regression). Quantitative variables were natural-log transformed and associated ORs correspond to an increase of 1 SD after standardisation, except for time from symptoms onset to hospital admission (1 day increase)

[a]eGFR determined by the CKD-EPI formula

Abbreviations: ALT, alanine aminotransferase; ULN, upper limit of normal; GGT, γ-glutamyl transferase; LDH, lactate dehydrogenase; CPK, creatine phosphokinase

**ESM Table 4 Multivariable analysis of the composite outcome within 7 days – full model**

| | Complete case population (337/1355 events, 24.9%) | | Complete case population with positive PCR (321/1249 events, 25.7%) | | Complete case population with RPG (112/761 events, 25.2%) | |
|---|---|---|---|---|---|---|
| **Variable** | **OR (95% CI)** | ***P* value** | **OR (95% CI)** | ***P* value** | **OR (95% CI)** | ***P* value** |
| Sex (female/male) | 0.78 (0.57, 1.06) | 0.11 | 0.81 (0.58, 1.11) | 0.19 | 0.75 (0.49, 1.15) | 0.18 |
| Age (+1 SD) | 0.85 (0.72, 1.01) | 0.057 | 0.85 (0.71, 1.01) | 0.060 | 0.88 (0.69, 1.11) | 0.28 |
| BMI (+1 SD) | 1.15 (0.98, 1.34) | 0.077 | 1.17 (0.99, 1.37) | 0.066 | 1.14 (0.92, 1.41) | 0.24 |
| Hypertension | 1.31 (0.88, 1.95) | 0.19 | 1.19 (0.79, 1.80) | 0.40 | 1.00 (0.59, 1.68) | 0.99 |
| Microvascular complications | 1.11 (0.76, 1.64) | 0.58 | 1.16 (0.77, 1.74) | 0.47 | 1.12 (0.67, 1.88) | 0.66 |
| Macrovascular complications | 0.93 (0.67, 1.29) | 0.66 | 0.92 (0.66, 1.30) | 0.65 | 0.99 (0.63, 1.56) | 0.97 |
| COPD | 0.89 (0.56, 1.42) | 0.63 | 0.96 (0.60, 1.55) | 0.88 | 1.30 (0.69, 2.45) | 0.41 |
| Treated OSA | 1.06 (0.68, 1.66) | 0.81 | 1.24 (0.78, 1.98) | 0.36 | 0.69 (0.35, 1.38) | 0.30 |
| Metformin | 0.70 (0.52, 0.94) | 0.018 | 0.71 (0.52, 0.97) | 0.033 | 0.62 (0.42, 0.93) | 0.022 |
| Insulin | 0.90 (0.66, 1.22) | 0.50 | 0.92 (0.67, 1.28) | 0.64 | 0.71 (0.46, 1.09) | 0.12 |
| ARBs and/or ACE inhibitors and/or MRA | 0.99 (0.72, 1.37) | 0.96 | 1.02 (0.73, 1.43) | 0.89 | 1.11 (0.71, 1.74) | 0.64 |
| Statins | 1.54 (1.14, 2.07) | 0.005 | 1.67 (1.22, 2.29) | 0.0013 | 1.44 (0.96, 2.16) | 0.082 |
| Anticoagulation therapy | 0.89 (0.61, 1.32) | 0.58 | 0.87 (0.58, 1.30) | 0.49 | 0.80 (0.45, 1.41) | 0.44 |
| Time from symptom onset to hospital admission (+1 SD) | 0.90 (0.75, 1.09) | 0.28 | 0.92 (0.76, 1.13) | 0.43 | 0.79 (0.61, 1.03) | 0.079 |
| Dyspnoea | 2.40 (1.74, 3.31) | <0.001 | 2.43 (1.74, 3.41) | <0.001 | 3.01 (1.91, 4.75) | <0.001 |
| eGFR (+1 SD) [a] | 0.93 (0.77, 1.11) | 0.41 | 0.95 (0.79, 1.15) | 0.60 | 0.92 (0.72, 1.19) | 0.55 |
| AST (% ULN) (+1 SD) | 1.39 (1.20, 1.61) | <0.001 | 1.43 (1.22, 1.67) | <0.001 | 1.44 (1.17, 1.76) | <0.001 |
| White cell count (+1 SD) | 1.17 (1.00, 1.37) | 0.050 | 1.12 (0.95, 1.32) | 0.19 | 1.00 (0.80, 1.24) | 0.97 |
| Platelet (+1 SD) | 0.72 (0.62, 0.84) | <0.001 | 0.70 (0.60, 0.82) | <0.001 | 0.75 (0.61, 0.94) | 0.011 |
| CRP (+1 SD) | 2.26 (1.85, 2.77) | <0.001 | 2.48 (1.99, 3.08) | <0.001 | 2.26 (1.72, 2.98) | <0.001 |
| Admission plasma glucose (+1 SD) | | | | | 1.46 (1.20, 1.77) | <0.001 |

Microvascular complication was defined as history of one or more of the following: diabetic kidney disease and/or severe diabetic retinopathy and/or history of diabetic foot ulcer. Macrovascular complication was defined as history of one or more of the following comorbidities: ischemic heart disease (acute coronary syndrome and/or coronary artery disease revascularisation) and/or cerebrovascular disease (stroke and/or transient ischaemic attack) and/or peripheral heart disease (amputation owing to ischemic disease and/or lower limb artery revascularisation)

[a]eGFR determined by the CKD-EPI formula

Abbreviations: COPD, Chronic Obstructive Pulmonary Disease; OSA, Obstructive sleep apnea; ARB, angiotensin-2 receptor blocker; ULN, upper limit of normal.

**ESM Table 5 COVID-19-related clinical outcomes within the first 7 and 28 days following hospital admission**

| | **CORONADO population, N = 2796** | | |
|---|---|---|---|
| | **Within 7 days** | **Between day 8 and day 28** | **Within 28 days** |
| Primary outcome: number (%, 95% CI) | 800 (28.6%, 26.9-30.3) | 179 (6.4%, 5.5-7.4) | 979 (35.0%, 33.2-36.8) |
| Death: number (%, 95% CI) | 312 (11.2%, 10.0-12.4) | 265 (9.5%, 8.4-10.6) | 577 (20.6%, 19.2-22.2) |
| Tracheal intubation: number (%, 95% CI) | 532 (19.0%, 17.6-20.5) | 24 (0.9%, 0.6-1.3) | 556 (19.9%, 18.4-21.4) |
| Admission to ICU: number (%, 95% CI) | 798 (28.5%, 26.9-30.3) | 25 (0.9%, 0.6-1.3) | 823 (29.4%, 27.7-31.2) |
| All discharges: number (%, 95% CI) | 704 (25.2%, 23.6-26.8) | 1173 (42.0%, 40.1-43.8) | 1877 (67.1%, 65.3-68.9) |
| Home discharge (home or previous long-term care facility): number (%, 95% CI) | 574 (20.5%, 19.0-22.1) | 830 (29.7%, 28.0-31.4) | 1404 (50.2%, 48.3-52.1) |
| Transfer – other hospital and/or rehabilitation care (%, 95% CI) | 130 (4.6%, 3.9-5.5) | 343 (12.3%, 11.1-13.5) | 473 (16.9%, 15.5-18.4) |

Population size was N= 2796. Data shown are *n* (%), and 95% CI of proportions are calculated using the Clopper-Pearson estimate.

A patient who presented successively tracheal intubation and death was counted only once for the primary outcome, which explain the greater number of death than primary outcome between day 8 and 28.

**ESM Table 6 Multivariable analysis of discharge and death within 28 days in complete case population, with raw features before transformation**

| | Overall population, N = 2796 | | Population analyzed in multivariable regression, N = 1355 | | | | |
|---|---|---|---|---|---|---|---|
| | | | | Discharge within 28 days (728/1355 events, 53.7%) | | Death within 28 days (225/1355 events, 16.6%) | |
| **Variable** | **Data available** | ***n* (%) or mean ± SD** | **N (%) or mean ± SD** | **OR (95% CI)** | ***P* value** | **OR (95% CI)** | ***P* value** |
| Sex (female/male) | 2796 | 1014 (36.3) | 503 (37.1) | 1.16 (0.90, 1.50) | 0.26 | 0.86 (0.60, 1.22) | 0.40 |
| Age (years) (+5) | 2796 | 69.7 ± 13.2 | 68.9 ± 13.2 | 0.87 (0.82, 0.92) | <0.001 | 1.23 (1.14, 1.33) | <0.001 |
| BMI (kg/m²) (+1) | 2460 | 29.2 ± 6.0 | 29.2 ± 5.8 | 1.00 (0.98, 1.02) | 0.88 | 1.01 (0.98, 1.04) | 0.56 |
| Hypertension | 2769 | 2126 (76.8) | 1042 (76.9) | 0.91 (0.65, 1.27) | 0.58 | 0.75 (0.46, 1.23) | 0.26 |
| Microvascular complications | 1966 | 869 (44.2) | 577 (42.6) | 0.76 (0.55, 1.05) | 0.094 | 1.92 (1.21, 3.05) | 0.006 |
| Macrovascular complications | 2627 | 1014 (38.6) | 518 (38.2) | 0.98 (0.75, 1.29) | 0.88 | 1.07 (0.75, 1.52) | 0.70 |
| COPD | 2732 | 263 (9.6) | 137 (10.1) | 1.00 (0.67, 1.50) | 0.98 | 1.03 (0.64, 1.67) | 0.90 |
| Treated OSA | 2593 | 273 (10.5) | 148 (10.9) | 1.27 (0.85, 1.88) | 0.24 | 0.91 (0.55, 1.53) | 0.73 |
| Metformin | 2794 | 1553 (55.6) | 737 (54.4) | 1.31 (1.02, 1.68) | 0.035 | 0.69 (0.48, 0.98) | 0.038 |
| Insulin | 2796 | 1039 (37.2) | 570 (42.1) | 0.82 (0.64, 1.07) | 0.14 | 1.34 (0.95, 1.91) | 0.010 |
| ARBs and/or ACE inhibitors and/or MRA | 2794 | 1570 (56.2) | 775 (57.2) | 1.11 (0.84, 1.45) | 0.47 | 0.94 (0.66, 1.34) | 0.72 |
| Statins | 2794 | 1282 (45.9) | 657 (48.5) | 0.82 (0.64, 1.06) | 0.13 | 1.48 (1.05, 2.09) | 0.027 |
| Anticoagulation therapy | 2794 | 501 (17.9) | 247 (18.2) | 0.64 (0.46, 0.88) | 0.006 | 1.10 (0.75, 1.62) | 0.62 |

**ESM Table 6 Multivariable analysis of discharge and death within 28 days in complete case population, with raw features before transformation (continued)**

| | Overall population, N = 2796 | | Population analyzed in multivariable regression, N = 1355 | | | | |
|---|---|---|---|---|---|---|---|
| | | | | Discharge within 28 days (728/1355 events, 53.7%) | | Death within 28 days (225/1355 events, 16.6%) | |
| **Variable** | **data available** | **n (%) or mean ± SD** | **n (%) or mean ± SD** | **OR (95% CI)** | ***P* value** | **OR (95% CI)** | ***P* value** |
| Time from symptom onset to hospital admission (day) (+1) | 2743 | 6.0 ± 5.4 | 6.1 ± 5.2 | 1.02 (1.00, 1.05) | 0.040 | 0.96 (0.92, 0.99) | 0.011 |
| Dyspnoea | 2754 | 1771 (64.3) | 864 (63.8) | 0.67 (0.52, 0.85) | 0.001 | 2.07 (1.44, 2.96) | <0.001 |
| eGFR (ml $min^{-1}$ $[1.73\ m]^{-2}$) [a] (+5) | 2602 | 66.1 ± 30.7 | 66.9 ± 30.6 | 1.02 (0.99, 1.05) | 0.12 | 0.95 (0.91, 0.99) | 0.015 |
| AST (% ULN) (+100%) | 2444 | 1.53 ± 3.24 | 1.38 ± 1.63 | 0.81 (0.72, 0.92) | 0.001 | 1.13 (1.03, 1.23) | 0.011 |
| White cell count ($10^3/mm^3$) (+1000) | 2726 | 7575 ± 5299 | 7405 ± 5751 | 0.95 (0.92, 0.99) | 0.006 | 1.04 (1.01, 1.07) | 0.006 |
| Platelet ($10^3/mm^3$) (+10) | 2725 | 217 ± 91 | 217 ± 91 | 1.01 (0.99, 1.04) | 0.30 | 0.98 (0.95, 1.02) | 0.36 |
| CRP (mg/L) (+10) | 2612 | 112 ± 147 | 109 ± 159 | 0.98 (0.96, 1.00) | 0.057 | 1.02 (1.00, 1.03) | 0.058 |

Models were applied to 1355 participants yielding 728 discharges (53.7%) and 225 deaths within 28 days (16.6%).

Microvascular complication was defined as history of one or more of the following: diabetic kidney disease and/or severe diabetic retinopathy and/or history of diabetic foot ulcer. Macrovascular complication was defined as history of one or more of the following comorbidities: ischemic heart disease (acute coronary syndrome and/or coronary artery disease revascularisation) and/or cerebrovascular disease (stroke and/or transient ischaemic attack) and/or peripheral heart disease (amputation owing to ischemic disease and/or lower limb artery revascularisation)

[a]eGFR determinted by the CKD-EPI formula

Abbreviations: COPD, Chronic Obstructive Pulmonary Disease; OSA, Obstructive sleep apnea; ARB, angiotensin-2 receptor blocker; ULN, upper limit of normal.

**ESM Table 7 Multivariable analysis of discharge within 28 days, complete case population and restricted to CORONADO participants with positive PCR or available admission plasma glucose: covariates prior to and on admission**

| | Complete case population (728/1355 events, 53.7%) | | Complete case population with positive PCR (666/1249 events, 53.3%) | | Complete case population with RPG (429/761 events, 56.4%) | |
|---|---|---|---|---|---|---|
| **Variable** | **OR (95% CI)** | ***P* value** | **OR (95% CI)** | ***P* value** | **OR (95% CI)** | ***P* value** |
| Sex (female/male) | 1.06 (0.82, 1.38) | 0.65 | 1.13 (0.86, 1.48) | 0.37 | 1.22 (0.86, 1.74) | 0.26 |
| Age (+1 SD) | 0.67 (0.58, 0.77) | <0.001 | 0.62 (0.54, 0.72) | <0.001 | 0.67 (0.55, 0.82) | <0.001 |
| BMI (+1 SD) | 1.01 (0.88, 1.16) | 0.89 | 0.98 (0.85, 1.13) | 0.77 | 1.00 (0.83, 1.21) | 0.98 |
| Hypertension | 0.88 (0.63, 1.24) | 0.47 | 0.88 (0.62, 1.25) | 0.47 | 0.93 (0.60, 1.46) | 0.76 |
| Microvascular complications | 0.70 (0.51, 0.97) | 0.031 | 0.66 (0.47, 0.92) | 0.014 | 0.79 (0.52, 1.21) | 0.28 |
| Macrovascular complications | 0.92 (0.70, 1.22) | 0.58 | 0.92 (0.69, 1.23) | 0.59 | 0.86 (0.59, 1.26) | 0.44 |
| COPD | 1.02 (0.68, 1.53) | 0.93 | 1.00 (0.65, 1.52) | 0.99 | 0.93 (0.53, 1.62) | 0.79 |
| Treated OSA | 1.23 (0.82, 1.83) | 0.32 | 1.02 (0.67, 1.55) | 0.93 | 1.52 (0.84, 2.74) | 0.16 |
| Metformin | 1.40 (1.08, 1.81) | 0.011 | 1.44 (1.10, 1.88) | 0.008 | 1.42 (1.00, 2.01) | 0.047 |
| Insulin | 0.80 (0.62, 1.04) | 0.091 | 0.76 (0.58, 1.00) | 0.053 | 0.84 (0.59, 1.19) | 0.33 |
| ARBs and/or ACE inhibitors and/or MRA | 1.09 (0.83, 1.44) | 0.52 | 1.03 (0.77, 1.38) | 0.84 | 1.01 (0.69, 1.48) | 0.95 |
| Statins | 0.83 (0.65, 1.07) | 0.16 | 0.87 (0.66, 1.13) | 0.29 | 0.83 (0.59, 1.18) | 0.30 |
| Anticoagulation therapy | 0.62 (0.45, 0.85) | 0.003 | 0.63 (0.45, 0.88) | 0.006 | 0.52 (0.33, 0.82) | 0.005 |
| Time from symptom onset to hospital admission (+1 SD) | 1.26 (1.08, 1.47) | 0.003 | 1.27 (1.07, 1.49) | 0.005 | 1.20 (0.98, 1.47) | 0.071 |
| Dyspnoea | 0.71 (0.55, 0.91) | 0.008 | 0.70 (0.54, 0.91) | 0.009 | 0.62 (0.44, 0.89) | 0.008 |
| eGFR (+1 SD) [a] | 1.05 (0.89, 1.22) | 0.57 | 0.98 (0.83, 1.15) | 0.78 | 1.07 (0.86, 1.33) | 0.53 |
| AST (% ULN) (+1 SD) | 0.72 (0.63, 0.84) | <0.001 | 0.73 (0.63, 0.85) | <0.001 | 0.70 (0.57, 0.85) | <0.001 |
| White cell count (+1 SD) | 0.83 (0.73, 0.95) | 0.008 | 0.83 (0.72, 0.96) | 0.013 | 0.83 (0.68, 1.01) | 0.062 |
| Platelet (+1 SD) | 1.07 (0.94, 1.22) | 0.29 | 1.07 (0.94, 1.23) | 0.31 | 1.01 (0.83, 1.22) | 0.94 |
| CRP (+1 SD) | 0.75 (0.66, 0.86) | <0.001 | 0.76 (0.66, 0.88) | <0.001 | 0.74 (0.62, 0.90) | 0.002 |
| Admission plasma glucose (+1 SD) | | | | | 0.82 (0.69, 0.98) | 0.028 |

Microvascular complication was defined as history of one or more of the following: diabetic kidney disease and/or severe diabetic retinopathy and/or history of diabetic foot ulcer. Macrovascular complication was defined as history of one or more of the following comorbidities: ischemic heart disease (acute coronary syndrome and/or coronary artery disease revascularisation) and/or cerebrovascular disease (stroke and/or transient ischaemic attack) and/or peripheral heart disease (amputation owing to ischemic disease and/or lower limb artery revascularisation)

[a]eGFR determinted by the CKD-EPI formula

Abbreviations: COPD, Chronic Obstructive Pulmonary Disease; OSA, Obstructive sleep apnea; ARB, angiotensin-2 receptor blocker; ULN, upper limit of normal.

**ESM Table 8 Multivariable analysis of death within 28 days, complete case population and restricted to CORONADO participants with positive PCR or available admission plasma glucose: covariates prior to and on admission**

| | Complete case population (225/1355 events, 16.6%) | | Complete case population with positive PCR (209/1249 events, 16.7%) | | Complete case population with RPG (112/761 events, 14.7%) | |
|---|---|---|---|---|---|---|
| **Variable** | **OR (95% CI)** | ***P* value** | **OR (95% CI)** | ***P* value** | **OR (95% CI)** | ***P* value** |
| Sex (female/male) | 1.01 (0.70, 1.45) | 0.96 | 0.92 (0.63, 1.34) | 0.65 | 0.79 (0.46, 1.36) | 0.40 |
| Age (+1 SD) | 1.84 (1.49, 2.27) | <0.001 | 1.86 (1.49, 2.32) | <0.001 | 2.16 (1.55, 3.02) | <0.001 |
| BMI (+1 SD) | 1.03 (0.86, 1.25) | 0.72 | 1.04 (0.86, 1.27) | 0.67 | 1.21 (0.92, 1.59) | 0.17 |
| Hypertension | 0.84 (0.51, 1.39) | 0.50 | 0.74 (0.45, 1.23) | 0.25 | 0.69 (0.34, 1.39) | 0.30 |
| Microvascular complications | 2.11 (1.35, 3.27) | <0.001 | 2.23 (1.40, 3.54) | <0.001 | 1.78 (0.95, 3.35) | 0.072 |
| Macrovascular complications | 1.15 (0.80, 1.66) | 0.43 | 1.23 (0.85, 1.80) | 0.27 | 1.21 (0.71, 2.06) | 0.47 |
| COPD | 1.03 (0.63, 1.68) | 0.91 | 1.08 (0.65, 1.79) | 0.78 | 1.70 (0.83, 3.47) | 0.14 |
| Treated OSA | 1.01 (0.60, 1.70) | 0.97 | 1.18 (0.69, 2.01) | 0.55 | 0.47 (0.19, 1.20) | 0.11 |
| Metformin | 0.65 (0.45, 0.93) | 0.020 | 0.62 (0.42, 0.90) | 0.013 | 0.66 (0.39, 1.11) | 0.12 |
| Insulin | 1.44 (1.01, 2.06) | 0.046 | 1.35 (0.92, 1.97) | 0.12 | 1.20 (0.71, 2.04) | 0.50 |
| ARBs and/or ACE inhibitors and/or MRA | 0.92 (0.64, 1.32) | 0.64 | 0.91 (0.62, 1.33) | 0.62 | 0.98 (0.57, 1.68) | 0.92 |
| Statins | 1.42 (1.00, 2.02) | 0.053 | 1.41 (0.97, 2.04) | 0.071 | 1.80 (1.07, 3.04) | 0.026 |
| Anticoagulation therapy | 1.21 (0.81, 1.79) | 0.35 | 1.23 (0.82, 1.86) | 0.31 | 1.46 (0.81, 2.65) | 0.21 |
| Time from symptom onset to hospital admission (+1 SD) | 0.72 (0.57, 0.90) | 0.004 | 0.72 (0.56, 0.91) | 0.008 | 0.79 (0.58, 1.06) | 0.12 |
| Dyspnoea | 1.89 (1.31, 2.73) | <0.001 | 1.97 (1.33, 2.90) | <0.001 | 2.02 (1.16, 3.53) | 0.013 |
| eGFR (+1 SD) [a] | 0.85 (0.70, 1.03) | 0.093 | 0.88 (0.72, 1.07) | 0.21 | 0.77 (0.57, 1.02) | 0.071 |
| AST (% ULN) (+1 SD) | 1.47 (1.25, 1.74) | <0.001 | 1.48 (1.24, 1.77) | <0.001 | 1.67 (1.32, 2.13) | <0.001 |
| White cell count (+1 SD) | 1.30 (1.10, 1.54) | 0.003 | 1.30 (1.08, 1.56) | 0.006 | 1.46 (1.13, 1.88) | 0.004 |
| Platelet (+1 SD) | 0.84 (0.71, 1.00) | 0.047 | 0.81 (0.68, 0.97) | 0.022 | 0.96 (0.73, 1.26) | 0.75 |
| CRP (+1 SD) | 1.48 (1.21, 1.80) | <0.001 | 1.50 (1.21, 1.85) | <0.001 | 1.63 (1.22, 2.18) | 0.001 |
| Admission plasma glucose (+1 SD) | | | | | 1.44 (1.12, 1.84) | 0.0045 |

Microvascular complication was defined as history of one or more of the following: diabetic kidney disease and/or severe diabetic retinopathy and/or history of diabetic foot ulcer. Macrovascular complication was defined as history of one or more of the following comorbidities: ischemic heart disease (acute coronary syndrome and/or coronary artery disease revascularisation) and/or cerebrovascular disease (stroke and/or transient ischaemic attack) and/or peripheral heart disease (amputation owing to ischemic disease and/or lower limb artery revascularisation)

[a]eGFR determinted by the CKD-EPI formula

Abbreviations: COPD, Chronic Obstructive Pulmonary Disease; OSA, Obstructive sleep apnea; ARB, angiotensin-2 receptor blocker; ULN, upper limit of normal.

## ESM FIGURES

## ESM Fig. 1 Flow diagram

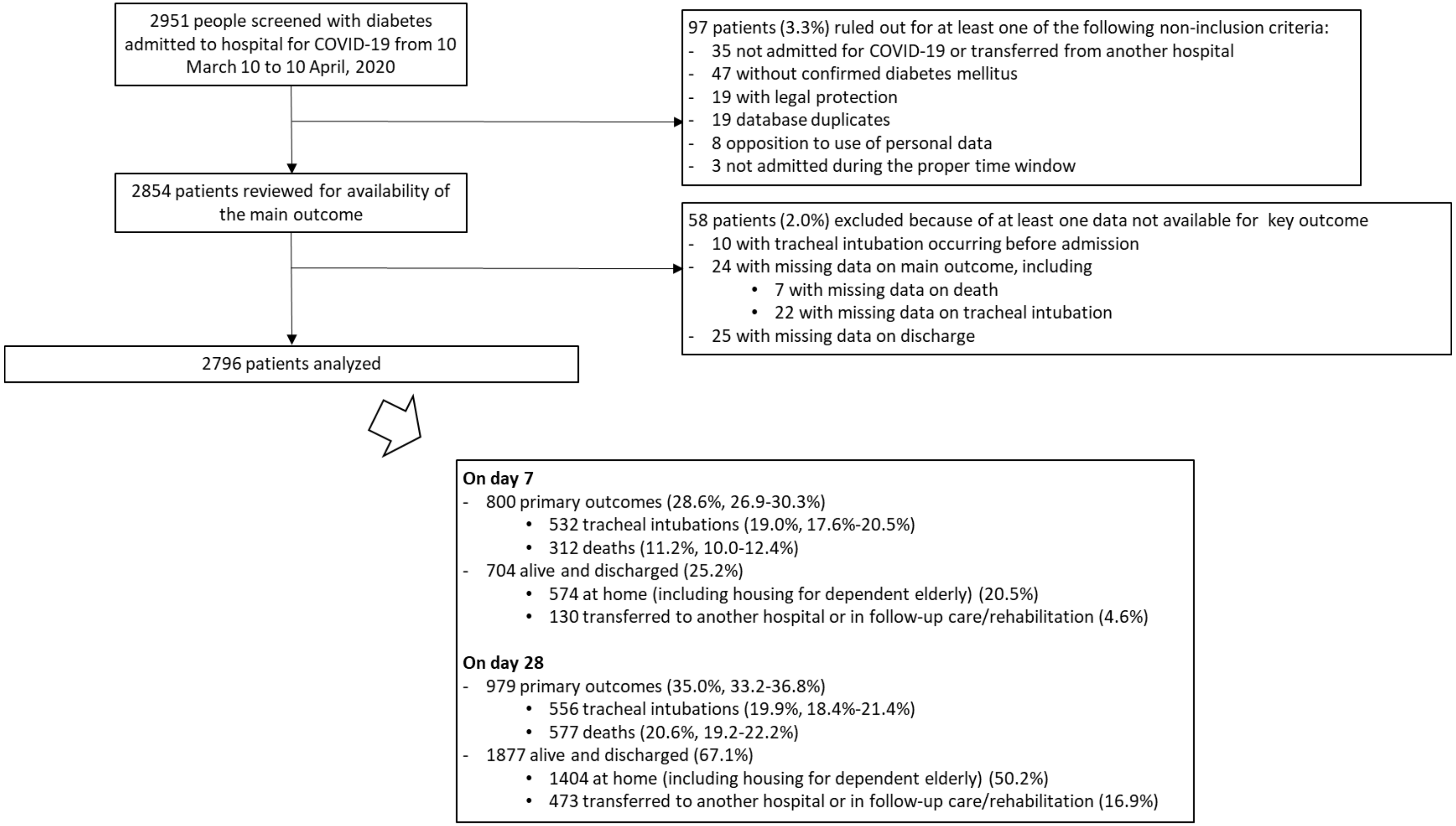


For proportions, 95% CI are calculated using the Clopper-Pearson estimate

**ESM Fig. 2a,b Time before discharge according to admission date, in individuals who went back home within 28 days**

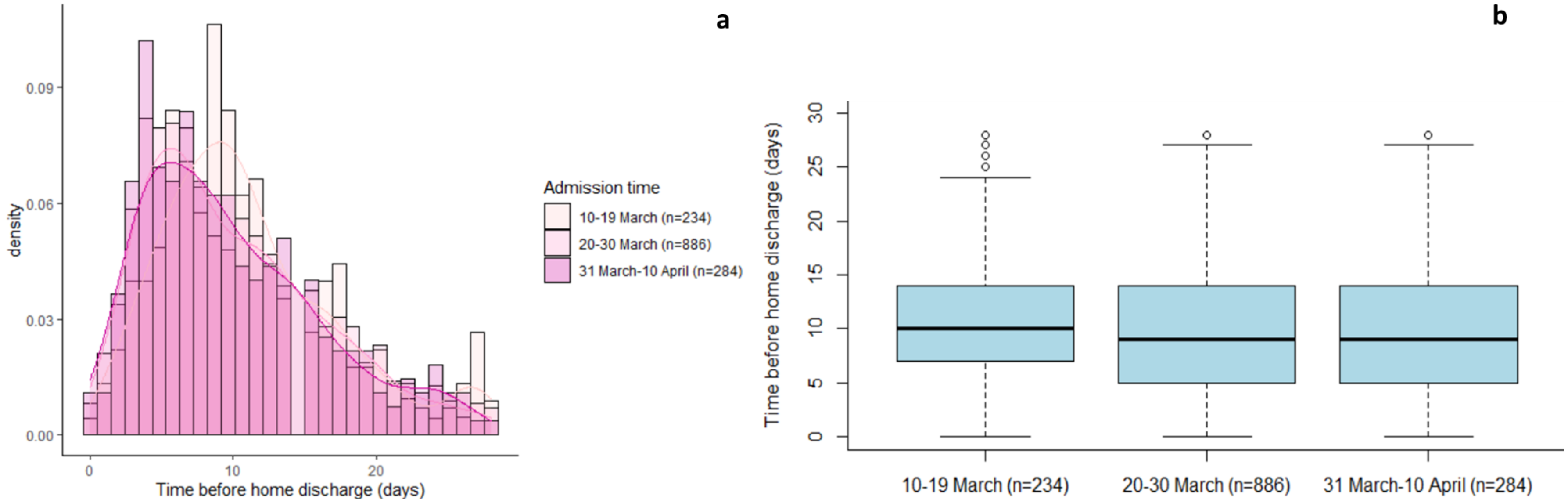


Population size was N=1404. (a) Frequency distribution of individuals according to time before discharge and 3 periods of admission date (10-19 March, 20-30 March and 31 March to 10 April). (b) Boxplot of time before home discharge according to 3 periods of admission date (10-19 March, 20-30 March and 31 March to 10 April)

**ESM TEXT**

**ESM Text 1 List of the collaborators of the CORONADO study**

CORONADO Scientific committee –

Pr. Bertrand Cariou (Nantes) : Principal Investigator
Pr. Samy Hadjadj (Nantes) : Scientific Coordinator
Pr. Bernard Bauduceau (Begin hospital, St Mandé) – Treasurer FFRD : scientific and medical expert (diabetology, gerontology)
Pr. David Boutoille (Nantes) : scientific and medical expert (infectious diseases)
Pr. France Cazenave-Roblot (Poitiers) – Vice-president SPILF : scientific and medical expert (infectious diseases)
Pr. Jean-François Gautier (Lariboisière hospital, Paris) – Vice-Président SFD : scientific and medical expert (diabetology)
Pr. Pierre Gourdy (Toulouse) scientific and medical expert (diabetology)
Pr. Véronique Kerlan (Brest) – President SFE : scientific and medical expert (diabetology)
Pr. Bruno Laviolle (Rennes) : scientific and medical expert (methodology)
Pr. René Robert (Poitiers) : scientific and medical expert (intensive care)
Pr. Ronan Roussel (Bichat hospital, Paris) : scientific and medical expert (diabetology)
Pr. Charles Thivolet (Lyon) – President SFD : scientific and medical expert (diabetology)
Dr. Matthieu Wargny (Nantes) : scientific and medical expert (methodology)
Dr. Matthieu Pichelin (Nantes) : Project manager – Scientific Coordination
Mr. Claude Chaumeil - Vice-Président Fédération Française des Diabétiques (FFD)
Mr. Jean François Thebaut Vice-Président Fédération Française des Diabétiques (FFD)

CORONADO Collaborators list –

Name, FAMILY NAME, Hospital, Town, Country, Email address

Matthieu, WARGNY, CHU de Nantes, Nantes, France, matthieu.wargny@chu-nantes.fr

Pascale, MAHOT, CHU de Nantes, Nantes, France, pascale.moreau@chu-nantes.fr

Bertrand, CARIOU, CHU de Nantes, Nantes, France, bertrand.cariou@univ-nantes.fr

Samy, HADJADJ, CHU de Nantes, Nantes, France, samy.hadjadj@univ-nantes.fr

Matthieu, PICHELIN, CHU de Nantes, Nantes, France, matthieu.pichelin@univ-nantes.fr

Anne-Laure, FOURNIER-GUILLOUX, CHU de Nantes, Nantes, France, annelaure.fournierguilloux@chu-nantes.fr

Nicolas, MAUDUIT, CHU de Nantes, Nantes, France, nicolas.mauduit@chu-nantes.fr

Edith, BIGOT- CORBEL, CHU de Nantes, Nantes, France, edith.bigot@chu-nantes.fr

Anne-Sophie, BOUREAU, CHU de Nantes, Nantes, France, annesophie.boureau@chu-nantes.fr

Laure, DE DEKCER, CHU de Nantes, Nantes, France, laure.dedecker@chu-nantes.fr

Audrey, ERNOULD, CHU de Nantes, Nantes, France, audrey.ernould@chu-nantes.fr

Claire, PRIMOT, CHU de Nantes, Nantes, France, claire.primot@chu-nantes.fr

Anne, SEGUIN, CHU de Nantes, Nantes, France, anne.seguin@chu-nantes.fr

Marielle, JOLIVEAU, CHU de Nantes, Nantes, France, marielle.joliveau@chu-nantes.fr

Sonia, POUVREAU, CHU de Nantes, Nantes, France, sonia.pouvreau@chu-nantes.fr

Chloé, FOURNIER, CHU de Nantes, Nantes, France, chloe.fournier@chu-nantes.fr

Jeremy, THUREAU, CHU de Nantes, Nantes, France, jeremy.thureau@chu-nantes.fr

Edith, FONTENEAU, CHU de Nantes, Nantes, France, edith.fonteneau@chu-nantes.fr

Pamela, HUBLAIN, CHU de Nantes, Nantes, France, Pamela.HUBLAIN@chu-nantes.fr

Carole, AGASSE, CHU de Nantes, Nantes, France, Carole.AGASSE@chu-nantes.fr

Mathilde, DE KERGARADEC, CHU de Nantes, Nantes, France, laurence.dekergaradec@chu-nantes.fr

Vincent, MINVILLE, CHU Toulouse, Toulouse, France, minville.v@chu-toulouse.fr

Fanny, VARDON-BOUNES, CHU Toulouse, Toulouse, France, bounes.f@chu-toulouse.fr

Guillaume, MARTIN-BLONDEL, CHU Toulouse, Toulouse, France, martin-blondel.g@chu-toulouse.fr

Pierre, GOURDY, CHU Toulouse, Toulouse, France, pierre.gourdy@inserm.fr

Blandine, TRAMUNT, CHU Toulouse, Toulouse, France, blandine.tramunt@orange.fr

Marie-Christine, TURNIN, CHU Toulouse, Toulouse, France, turnin.mc@chu-toulouse.fr

Hélène, HANAIRE, CHU Toulouse, Toulouse, France, hanaire.h@chu-toulouse.fr

Jean-Michel, MANSUY, CHU Toulouse, Toulouse, France, mansuy.jm@chu-toulouse.fr

Didier, FABRE, CHU Toulouse, Toulouse, France, fabre.d@chu-toulouse.fr

Marie-Blanche, ARHAINX, CHU Toulouse, Toulouse, France, arhainx.mb@chu-toulouse.fr

Laurent, CAZALS, CHU Toulouse, Toulouse, France, cazals.l@chu-toulouse.fr

Laure, COMBES, CHU Toulouse, Toulouse, France, combes.l@chu-toulouse.fr

Emmanuelle, LAMI, CHU Toulouse, Toulouse, France, lami.e@chu-toulouse.fr

Mallory, CIANFERANI, CHU Toulouse, Toulouse, France, cianferani.m@chu-toulouse.fr

Bruno, MEGARBANE, Hôpital LARIBOISIERE, Paris, France, bruno.megarbane@aphp.fr

Pierre, LEROY, Hôpital LARIBOISIERE, Paris, France, pierre.leroy@aphp.fr

Jean-François, GAUTIER, Hôpital LARIBOISIERE, Paris, France, jean-francois.gautier@aphp.fr

Tiphaine, VIDAL-TRECAN, Hôpital LARIBOISIERE, Paris, France, tiphaine.vidal-trecan@aphp.fr

Jean-Pierre, RIVELINE, Hôpital LARIBOISIERE, Paris, France, jeanpierre.riveline@aphp.fr

Jean-Louis, LAPLANCHE, Hôpital LARIBOISIERE, Paris, France, jean-louis.laplanche@aphp.fr

Stéphane, MOULY, Hôpital LARIBOISIERE, Paris, France, stephane.mouly@aphp.fr

Louis, POTIER, Hôpital BICHAT, Paris, France, louis.potier@gmail.com

Ronan, ROUSSEL, Hôpital BICHAT, Paris, France, ronan.roussel@aphp.fr

Malak, TAHER, Hôpital BICHAT, Paris, France, malak.taher@aphp.fr

Yawa, ABOULEKA, Hôpital BICHAT, Paris, France, yawa.abouleka@aphp.fr

Fetta, YAKER, Hôpital BICHAT, Paris, France, fettaamel.yaker@aphp.fr

Aurelie, CARLIER, Hôpital BICHAT, Paris, France, aurelie.carlier@aphp.fr

Anne, BOUTTEN, Hôpital BICHAT, Paris, France, anne.boutten@aphp.fr

Marilyne, HALLOT-FERON, Hôpital BICHAT, Paris, France, marilyne.feron@aphp.fr

Fadila, MOURAH, Hôpital BICHAT, Paris, France, fadila.mourah@gmail.com

Charles, THIVOLET, Hôpital Lyon Sud, Pierre Bénite, France, charles.thivolet@chu-lyon.fr

Emilie, BLOND, Hôpital Lyon Sud, Pierre Bénite, France, emilie.blond@chu-lyon.fr

Muriel, ROLLAND, Hôpital Lyon Sud, Pierre Bénite, France, muriel.rolland@chu-lyon.fr

Josep, VERDECHO MENDEZ, Hôpital Lyon Sud, Pierre Bénite, France, josep.verdecho-mendez@chu-lyon.fr

Marine, ALEXANDRE, Hôpital Lyon Sud, Pierre Bénite, France, marine.alexandre@chu-lyon.fr

Julien, POTTECHER, Nouvel hôpital civil, Strasbourg, France, julien.pottecher@chru-strasbourg,fr

Emilie, RICHER, Nouvel hôpital civil, Strasbourg, France, emilie.richerdupont@chru-strasbourg.fr

Laurent, MEYER, Nouvel hôpital civil, Strasbourg, France, laurent.meyer@chru-strasbourg.fr

Florina, LUCA, Hôpital Hautepierre, Strasbourg, France, florina.luca@chru-strasbourg,fr

Jean-Marc, LESSINGER, Nouvel hôpital civil, Strasbourg, France, Jean-Marc.LESSINGER@chru-strasbourg.fr

Thibault, BAHOUGNE, Hospices civils, Strasbourg, France, thibault.bahougne@chru-strasbourg,fr

Bruno, GUERCI, CHU Brabois Université de Lorraine, Nancy, France, b.guerci@chru-nancy.fr

Lisa, LUDWIG, CHU Brabois Université de Lorraine, Nancy, France, L.LUDWIG@chru-nancy.fr

Siham, BENZIRAR, CHU Brabois Université de Lorraine, Nancy, France, s.benzirar@chru-nancy.fr

Catherine, MALAPLATE, CHU Brabois Université de Lorraine, Nancy, France, c.malaplate@chu-nancy.fr

Thierry, MATTON, CHU Brabois Université de Lorraine, Nancy, France, t.matton@chru-nancy.fr

Julien, POISSY, Hôpital salengro CHU, Lille, France, julien.poissy@chru-lille.fr

Karine, FAURE, Hôpital Huriez CHRU, Lille, France, karine.faure@chru-lille.fr

Pierre, FONTAINE, Hôpital Huriez CHRU, Lille, France, pierre.fontaine@chru-lille.fr

Florence, BAUDOUX, Hôpital Huriez CHRU, Lille, France, florence.baudoux@chru-lille.fr

Anne, VAMBERGUE, Hôpital Huriez CHRU, Lille, France, anne.vambergue@chru-lille.fr

Jean David, PEKAR, Hôpital Huriez CHRU, Lille, France, jeandavid.PEKAR@chru-lille.fr

Marc, LAMBERT, Hôpital Calmette CHU, Lille, France, marc.lambert@chru-lille.fr

Cécile, YELNIK, Hôpital Calmette CHU, Lille, France, cecile.yelnik@chru-lille.fr

Amélie, BRUANDET, Hôpital Huriez CHRU, Lille, France, amelie.bruandet@chru-lille.fr

Laurent, PETIT, CHU de Bordeaux, Bordeaux, France, laurent.petit@chu-bordeaux.fr

Didier, NEAU, CHU de Bordeaux, Bordeaux, France, didier.neau@chu-bordeaux.fr

Vincent, RIGALLEAU, CHU de Bordeaux, Pessac, France, vincent.rigalleau@chu-bordeaux.fr

Annie, BERARD, CHU de Bordeaux, Bordeaux, France, annie.berard@chu-Bordeaux.fr

Amandine, GALIOOT, CHU de Bordeaux, Pessac, France, amandine.galioot@chu-bordeaux.fr

Remy, COUDROY, CHU Poitiers, Poitiers, France, Remi.COUDROY@chu-poitiers.fr

Arnaud, THILLE, CHU Poitiers, Poitiers, France, arnaud.thille@chu-poitiers.fr

René, ROBERT, CHU Poitiers, Poitiers, France, rene.robert@chu-poitiers.fr

France, ROBLOT-CAZENAVE, CHU Poitiers, Poitiers, France, France.CAZENAVE-ROBLOT@chu-poitiers.fr

Blandine, RAMMAERT, CHU Poitiers, Poitiers, France, blandine.rammaert@chu-poitiers.fr

Pierre Jean, SAULNIER, CHU Poitiers, Poitiers, France, pierrejean.saulnier@gmail.com

Xavier, PIGUEL, CHU Poitiers, Poitiers, France, xavier.piguel@chu-poitiers.fr

Nesrine, BENHENDA, CHU Poitiers, Poitiers, France, Nesrine.BENHENDA@chu-poitiers.fr

Camille, HUSSON, CHU Poitiers, Poitiers, France, Camille.HUSSON@chu-poitiers.fr

Celine, OLIVIER, CHU Poitiers, Poitiers, France, celine.olivier@chu-poitiers.fr

Florence, TORREMOCHA, CHU Poitiers, Poitiers, France, florence.torremocha@chu-poitiers.fr

Mathilde, FRATY, CHU Poitiers, Poitiers, France, mathilde.fraty@chu-poitiers.fr

Marie, FLAMEN D'ASSIGNY, CHU Poitiers, Poitiers, France, marie.flamen-dassigny@chu-poitiers.fr

Aurelie, MIOT, CHU Poitiers, Poitiers, France, aurelie.miot@chu-poitiers.fr

Valentin, BOSSARD, CHU Poitiers, Poitiers, France, valentin.bossard988@gmail.com

Kada, KLOUCHE, Hôpital Lapeyronie, Chu Montpellier, France, k-klouche@chu-montpellier.fr

Alain, MAKINSON, Hôpital Lapeyronie, Chu Montpellier, France, a-makinson@chu-montpellier.fr

Ariane, SULTAN, Hôpital Lapeyronie, Chu Montpellier, France, a-sultan@chu-montpellier.fr

Jean-Baptiste, BONNET, Hôpital Lapeyronie, Chu Montpellier, France, jean-baptiste-bonnet@chu-montpellier.fr

Vincent, FOULONGNE, Hôpital St Eloi, Chu Montpellier, France, v-foulongne@chu-montpellier.fr

Florence, GALTIER, Hôpital St Eloi, Chu Montpellier, France, f-galtier@chu-montpellier.fr

Cécile, AUBRON, CHU de Brest, Brest, France, cecile.aubron@ch-brest.fr

Séverine, ANSART, CHU de Brest, Brest, France, severine.ansart@chu-brest.fr

Véronique, KERLAN, CHU de Brest, Brest, France, veronique.kerlan@chu-brest.fr

Pascale, QUINIOU, CHU de Brest, Brest, France, pascale.quiniou@chu-brest;fr

Jean- Luc, CARRE, CHU de Brest, Brest, France, jean-luc.carre@chu-brest.fr

Stéphane, QUESNOT, CHU de Brest, Brest, France, stephane.quesnot@chu-brest.fr

Bruno, LAVIOLLE, CHU de Rennes, Rennes, France, bruno.laviolle@chu-rennes.fr

Carole, SCHWEBEL, CHU Grenoble Alpes, Grenoble, France, Cschwebel@chu-grenoble.fr

Olivier, EPAULARD, CHU Grenoble Alpes, Grenoble, France, OEpaulard@chu-grenoble.fr

Pierre-Yves, BENHAMOU, CHU Grenoble Alpes, Grenoble, France, PYBenhamou@chu-grenoble.fr

Cécile, BETRY, CHU Grenoble Alpes, Grenoble, France, Cbetry@chu-grenoble.fr

Anne-Laure, BOREL, CHU Grenoble Alpes, Grenoble, France, ALBorel@chu-grenoble.fr

Sandrine, LABLANCHE, CHU Grenoble Alpes, Grenoble, France, Slablanche@chu-grenoble.fr

Dorra, GUERGOUR, CHU Grenoble Alpes, Grenoble, France, Dguergour@chu-grenoble.fr

Catherine, DUCLOS, Hôpital AVICENNE, Bobigny, France, catherine.duclos@aphp.fr

Emmanuel, COSSON, Hôpital AVICENNE, Bobigny, France, emmanuel.cosson@aphp.fr

Erwan, GUYOT, Hôpital AVICENNE, Bobigny, France, erwan.guyot@aphp.fr

Aurore, DENIAU, Hôpital AVICENNE, Bobigny, France, aurore.deniau@aphp.fr

Phucthutrang, NGUYEN, Hôpital AVICENNE, Bobigny, France, phucthutrang.nguyen@aphp.fr

Yves, REZNIK, CHU Caen Normandie, Caen, France, reznik-y@chu-caen.fr

Michael, JOUBERT, CHU Caen Normandie, Caen, France, joubert-m@chu-caen.fr

Stéphane, ALLOUCHE, CHU Caen Normandie, Caen, France, allouche-s@chu-caen.fr

Lydia, GUITTET, CHU Caen Normandie, Caen, France, guittet-l@chu-caen.fr

Steven, GRANGE, CHU Rouen, Rouen, France, steven.grange@chu-rouen.fr

Manuel, ETIENNE, CHU Rouen, Rouen, France, manuel.etienne@chu-rouen.fr

Gaëtan, PRÉVOST, CHU Rouen, Rouen, France, gaetan.prevost@chu-rouen.fr

Valéry, BRUNEL, CHU Rouen, Rouen, France, valery.brunel@chu-rouen.fr

Jean-Christophe, LAGIER, IHU Marseille, Marseille, France, JeanChristophe.LAGIER@ap-hm.fr

Didier, RAOULT, IHU Marseille, Marseille, France, didier.raoult@ap-hm.fr

Anne, DUTOUR, CHU Nord et Conception, Marseille, France, anne.dutour@ap-hm.fr

Bénédicte, GABORIT, CHU Nord et Conception, Marseille, France, benedicte.gaborit@ap-hm.fr

Sandrine, BOULLLU, CHU Nord, Marseille, France, sandrine.boullu@ap-hm.fr

Patrice, DARMON, CHU Nord et Conception, Marseille, France, patrice.darmon@ap-hm.fr

Adèle, LASBLEIZ, CHU Nord et Conception, Marseille, France, adele.lasbleiz@ap-hm.fr

Mathieu, CERINO, CHU Conception, Marseille, France, mathieu.cerino@ap-hm.fr

Fanny, ROMAIN, CHU Conception, Marseille, France, fanny.romain@ap-hm.fr

Marie, HOUSSAYS, CHU Conception, Marseille, France, marie.houssays@ap-hm.fr

Jean Pierre, QUENOT, CHU François Mitterand, Dijon, France, jean-pierre.quenot@chu-dijon.fr

Lionel, PIROTH, CHU François Mitterand, Dijon, France, lionel.piroth@chu-dijon.fr

Bruno, VERGÈS, CHU François Mitterand, Dijon, France, bruno.verges@chu-dijon.fr

Laurence, DUVILLARD, CHU François Mitterand, Dijon, France, laurence.duvillard@chu-dijon.fr

Bernard, BONNOTTE, CHU François Mitterand, Dijon, France, bernard.bonnotte@chu-dijon.fr

Alain, MERCAT, CHU ANGERS, Angers, France, almercat@chu-angers.fr

Vincent, DUBEE, CHU ANGERS, Angers, France, vincent.dubee@chu-angers.fr

Ingrid, ALLIX, CHU ANGERS, Angers, France, inallix@chu-angers.fr

Patrice, RODIEN, CHU ANGERS, Angers, France, parodien@chu-angers.fr

Robin, DHERSIN, CHU ANGERS, Angers, France, Robin.Dhersin@chu-angers.fr

Maylis, LEBEAULT, CHU ANGERS, Angers, France, maylis.lebeault@chu-angers.fr

wojciech, TRZEPIZUR, CHU ANGERS, Angers, France, WoTrzepizur@chu-angers.fr

Jocelyne, LOISON, CHU ANGERS, Angers, France, jocelyne.loison@chu-angers.fr

Antoine, BRANGIER, CHU ANGERS, Angers, France, antoine.brangier@chu-angers.fr

Pierre, ASFAR, CHU ANGERS, Angers, France, piasfar@chu-angers.fr

Pascal, REYNIER, CHU ANGERS, Angers, France, pareynier@chu-angers.fr

Françoise, LARCHER, CHU ANGERS, Angers, France, frlarcher@chu-angers.fr

Françoise, JOUBAUD, CHU ANGERS, Angers, France, frjoubaud@chu-angers.fr

Marie-Rita, ANDREU, CHU ANGERS, Angers, France, marierita.andreu@chu-angers.fr

Geoffrey, URBANSKI, CHU ANGERS, Angers, France, geoffrey.urbanski@chu-angers.fr

Laurent, HUBERT, CHU ANGERS, Angers, France, lahubert@chu-angers.fr

Cedric, ANNWEILER, CHU ANGERS, Angers, France, ceannweiler@chu-angers.fr

Jean, DELLAMONICA, CHU de Nice - Hôpital de l'Archet, Nice, France, dellamonica.j@chu-nice.fr

Johan, COURJON, CHU de Nice - Hôpital de l'Archet, Nice, France, courjon.j@chu-nice.fr

Nicolas, CHEVALIER, CHU de Nice - Hôpital de l'Archet, Nice, France, chevalier.n@chu-nice.fr

Giulia, CHINETTI, CHU de Nice - Hôpital Pasteur, Nice, France, chinetti.g@chu-nice.fr

Magda, CHAFAI, CHU de Nice - Hôpital de l'Archet, Nice, France, chafai.m@chu-nice.fr

Bruno, MOURVILLIER, CHU de Reims, Reims, France, bmourvillier@chu-reims.fr

Firouze, BANI-SADR, CHU de Reims, Reims, France, fbanisadr@chu-reims.fr

Sarra, BARRAUD, CHU de Reims, Reims, France, sbarraud@chu-reims.fr

Brigitte, DELEMER, CHU de Reims, Reims, France, bdelemer@chu-reims.fr

Philippe, GILLERY, CHU de Reims, Reims, France, pgillery@chu-reims.fr

Pascale, LABEDADE, Centre Hospitalier Sud-Francilien, Corbeil-Essonnes, France, pascale.labedade@chsf.fr

Amélie, CHABROL, Centre Hospitalier Sud-Francilien, Corbeil-Essonnes, France, amelie.chabrol@chsf.fr

Alfred, PENFORNIS, Centre Hospitalier Sud-Francilien, Corbeil-Essones, France, alfred.penfornis@chsf.fr

Catherine, PETIT, Centre Hospitalier Sud-Francilien, Corbeil-Essones, France, catherine.petit@chsf.fr

Coralie, AMADOU, Centre Hospitalier Sud-Francilien, Corbeil-Essonnes, France, coralie.amadou@chsf.fr

Maxime, ADLER, Centre Hospitalier Sud-Francilien, Corbeil-Essonnes, France, maxime.adler@chsf.fr

Clément, DUBOST, HIA Bégin, Saint Mandé, France, clement.dubost@intradef.gouv.fr

Pierre-Louis, CONAN, HIA Bégin, Saint Mandé, France, pierre-louis.conan@intradef.gouv.fr

Lyse, BORDIER, HIA Bégin, Saint Mandé, France, lyse.bordier@intradef.gouv.fr

Franck, CEPPA, HIA Bégin, Saint Mandé, France, franck.ceppa@intradef.gouv.fr

Cyril, GARCIA, HIA Bégin, Saint Mandé, France, cyril1.garcia@intradef.gouv.fr

Mathilde, SOLLIER, HIA Bégin, Saint Mandé, France, mathilde.sollier@intradef.gouv.fr

Olivier, DUPUY, GH Paris Saint Joseph, Paris, France, odupuy@hpsj.fr

Sophie, LAPLANCE, GH Paris Saint Joseph, Paris, France, slaplanche@hpsj.fr

Olivier, BILLUART, GH Paris Saint Joseph, Paris, France, obilluart@hpsj.fr

Marie Joseph, AROULANDA, GH Paris Saint Joseph, Paris, France, mjaroulanda@hpsj.fr

Frédérique, OLIVIER, CH CAHORS, Cahors, France, frederique.olivier@ch-cahors.fr

Florence, AYON, CH CAHORS, Cahors, France, florence.ayon@ch-cahors.fr

Nathalie, WILHELM, CH CAHORS, Cahors, France, nathalie.wilhelm@ch-cahors.fr

Loic, EPELBOIN, CHU Cayennes, Cayenne, France, loic.epelboin@ch-cayenne.fr

Nadia, SABBAH, CHU Cayennes, Cayenne, France, nadia.sabbah@ch-cayenne.fr

Aurelie, CHARPIN, CHU Cayennes, Cayenne, France, aurelie.charpin@ch-cayenne.fr

Pierre, SQUARA, Clinique Ambroise Paré, Paris, France, pierre.squara@orange.fr

Olivier, BELLIARD, Clinique Ambroise Paré, Paris, France, olivier_belliard@yahoo.fr

Claude, DUBOIS, Clinique Ambroise Paré, Paris, France, claude.dubois@clinique-a-pare.fr

Michel, MARRE, Clinique Ambroise Paré, Paris, France, marre.michel@gmail.com

Johann, AUCHABIE, CH Cholet, Cholet, France, johann.auchabie@ch-cholet.fr

Roxane, COURTOIS, CH Cholet, Cholet, France, roxane.courtois@ch-cholet.fr

Thierry, DURIEZ, CH Cholet, Cholet, France, thierry.duriez@ch-cholet.fr

Tiphaine, MERGEY, CH Cholet, Cholet, France, tiphaine.mergey@ch-cholet.fr

Laura, VALLEE, CH Cholet, Cholet, France, laura.vallee@ch-cholet.fr

Laetitia, SEGUIN, CH Cholet, Cholet, France, laetitia.seguin@ch-cholet.fr

Abdallah, AL-SALAMEH, CHU Amiens- Picardie, Amiens, France, al-salameh.abdallah@chu-amiens.fr

Jean-Philippe, LANOIX, CHU Amiens- Picardie, Amiens, France, lanoix.jean-philippe@chu-amiens.fr

Sandrine, SORIOT-THOMAS, CHU Amiens- Picardie, Amiens, France, soriot-thomas.sandrine@chu-amiens.fr

Anne-Marie, BOURGEOIS-DESCOULS, CHU Amiens- Picardie, Amiens, France, bourgeois.anne-marie@chu-amiens.fr

Rachel, DESAILLOUD, CHU Amiens- Picardie, Amiens, France, dessaillud.rachelàchu-amiens.fr

Natacha, GERMAIN, CHU de Saint Etienne, Saint Etienne, France, natacha.germain@chu-st-etienne.fr

Bogdan, GALUSCA, CHU de Saint Etienne, Saint Etienne, France, bogdan.galusca@chu-st-etienne.fr

Gwenaelle, BELLETON, CHU de Saint Etienne, Saint Etienne, France, gwenaelle.belleton@chu-st-etienne.fr

Nesrine, MAROUANI, CHU de Saint Etienne, Saint Etienne, France, nesrine.marouani@chu-st-etienne.fr

Delia, PALAGHIU, CHU de Saint Etienne, Saint Etienne, France, delia.palaghiu@chu-st-etienne.fr

Amira, HAMMOUR, CHU de Saint-Etienne, Saint-Etienne, France, amira.hammour@chu-st-etienne.fr

Fernando, BERDAGUER, Hôpital Nord Franche-Comté, Belfort, France, fberdaguer@hotmail.com

Thimothée, KLOPFENSTEIN, Hôpital Nord Franche-Comté, Belfort, France, Timothee.KLOPFENSTEIN@hnfc.fr

Hajer, ZAYET, Hôpital Nord Franche-Comté, Belfort, France, Hajer.ZAYET@hnfc.fr

Patrice, WINISZEWSKI, Hôpital Nord Franche-Comté, Belfort, France, Patrice.WINISZEWSKI@hnfc.fr

Marie, ZANUSSO, Hôpital Nord Franche-Comté, Belfort, France, marie.zanusso@hnfc.fr

Pauline, GARNIER, Hôpital Nord Franche-Comté, Belfort, France, pauline.garnier@hnfc.fr

Ingrid, JULIER, CH de Ales, Ales, France, dr.julier@ch-ales.fr

Karim, HAMZAOUI, CH de Ales, Ales, France, dr.hamzaoui@ch-ales.fr

Sophie, MARTY-GRES, CH de Ales, Ales, France, biologie@ch-ales.fr

Tarik, EL SADKI, CH de Ales, Ales, France, biologie@ch-ales.fr

Lucile, CADOT, CH de Ales, Ales, France, biologie@ch-ales.fr

Jean-Louis, DUBOST, CH de Pontoise, Pontoise, France, jean-louis.dubost@ght-novo.fr

Céline, GONFROY, CH de Pontoise, Pontoise, France, celine.gonfroy@ght-novo.fr

Catherine, CAMPINOS, CH de Pontoise, Pontoise, France, catherine.campinos@ght-novo.fr

Pascale, MARTRES, CH de Pontoise, Pontoise, France, pascale.martres@ght-novo.fr

Marie Pierre, COULHON, CH de Pontoise, Pontoise, France, marie-pierre.coulhon@ght-novo.fr

Nicolas, ALLOU, CHU Felix Guyon, Saint Denis, France, nicolas.allou@chu-reunion.fr

Marwa, BACHIR, CHU Felix Guyon, Saint Denis, France, marwa.bachir@chu-reunion.fr

Stella, HOANG, CHU Felix Guyon, Saint Denis, France, stella.hoang@chu-reunion.fr

Candice, KEMBELLEC, CHU Felix Guyon, Saint Denis, France, candice.kembellec@chu-reunion.fr

Olivia, SUPLY, CHU Felix Guyon, Saint Denis, France, olivia.suply@chu-reunion.fr

Fatima, KHARCHA, CHU Felix Guyon, Saint Denis, France, fatima.kharcha@chu-reunion.fr

Anne-Claire, DEVOUGE, CHU Felix Guyon, Saint Denis, France, anne-claire.devouge@chu-reunion.fr

Anna, FLAUS-FURMANUK, CHU Felix Guyon, Saint Denis, France, anna.flaus-furmaniuk@chu-reunion.fr

Isabelle, MADELINE, CHU Felix Guyon, Saint Denis, France, isabelle.madeline@chu-reunion.fr

Vincent, EHINGER, CHU Felix Guyon, Saint Denis, France, vincent.ehinger@chu-reunion.fr

Sophie, BASTARD, CHU Felix Guyon, Saint Denis, France, sophie.bastard@chu-reunion.fr

Loic, RAFFRAY, CHU Felix Guyon, Saint Denis, France, loic.raffray@chu-reunion.fr

Frederic, RENOU, CHU Felix Guyon, Saint Denis, France, frederic.renou@chu-reunion.fr

Aude, BOJARSKI, CHU Felix Guyon, Saint Denis, France, aude.bojarski@chu-reunion.fr

Caroline, PAUL, CHU Felix Guyon, Saint Denis, France, caroline.paul@chu-reunion.fr

Karine, BORSU, CHU Felix Guyon, Saint Denis, France, karine.borsu@chu-reunion.fr

Angelique, GORLIN, CHU Felix Guyon, Saint Denis, France, angelique.gorlin@chu-reunion.fr

Servane, DI BERNARDO, CHU Felix Guyon, Saint Denis, France, servane.dibernardo@chu-reunion.fr

Carole, TRUONG VAN UT, CHU Felix Guyon, Saint Denis, France, carole.truong-van-ut@chu-reunion.fr

Stephane, RENAUD, CHU Felix Guyon, Saint Denis, France, stephane.renaud@chu-reunion.fr

Antoine, VIGNOLES, CHU Felix Guyon, Saint Denis, France, antoine.vignoles@chu-reunion.fr

Emilie, FOCH, CHU Felix Guyon, Saint Denis, France, emilie.foch@chu-reunion.fr

Laurie, MASSE, CHU Felix Guyon, Saint Denis, France, laurie.masse@chu-reunion.fr

Hubert, GRAND, robert boulin, Libourne, France, hubert.grand@ch-libourne.fr

Helene, FERRAND, robert boulin, Libourne, France, helene.ferrand@ch-libourne.fr

Christelle, RAFFAITIN-CARDIN, robert boulin, Libourne, France, christelle.raffaitin@ch-libourne.fr

Hadjer, ZELLAGUI, robert boulin, Libourne, France, hadjer.zellagui@ch-libourne.fr

Celine, CASTANG-BRACHET, robert boulin, Libourne, France, celine.castang@ch-libourne.fr

Frederique, BOURY, robert boulin, Libourne, France, frederique.boury@ch-libourne.fr

Ana, ALVAREZ TENA, CH Albi, Albi, France, ana.alvareztena@ch-albi,fr

Isabelle, MOURA, CH Albi, Albi, France, isabelle.moura@ch-albi.fr

Pierre, KALFON, LOUIS PASTEUR, Le Coudray, France, pkalfon@ch-chartres.fr

Juliana, DARASTEANU, LOUIS PASTEUR, Le Coudray, France, jdarasteanu@ch-cahrtres.fr

Arnaud, MONIER, LOUIS PASTEUR, Le Coudray, France, amonier@ch-chartres.fr

Pascal, FOUCAULT, LOUIS PASTEUR, Le Coudray, France, pfoucault@ch-chartres.fr

Alexandra, DEPUILLE, LOUIS PASTEUR, Le Coudray, France, adepuille@ch-chartres.fr

Stéphanie, LAUGIER-ROBIOLLE, CH D'AUCH, 32000, France, s.laugier-robiolle@ch-auch.fr

Patrick, CANEIRO, CH D'AUCH, 32000, France, p.caneiro@ch-auch.fr

Maud, BASSO, CH D'AUCH, 32000, France, maud.basso@ch-auch.fr

Etienne, LARGER, Hôpital COCHIN, Paris, France, etienne.larger@aphp.fr

Samir, BOUAM, Hôpital COCHIN, Paris, France, samir.bouam@aphp.fr

Wahiba, BENZENATI, Hôpital COCHIN, Paris, France, wahiba.benzenati-ext@aphp.fr

Leila, AIT BACHIR, Hopital francobritannique, Levallois, France, leila.aitbachir@ihfb.org

Camille, CUSSAC PILLEGAND, Hopital francobritannique, Levallois, France, camille.cussac-pillegand@ihfb.org

Marc, VASSE, Hopital francobritannique, Suresnes, France, marc.vasse@hopital-foch.fr

Christophe, MICHARD, CH du Forez, Montbrison, France, christophe.michard@ch-forez.fr

Nathanaëlle, MONTANIER, CH du Forez, Montbrison, France, nathanaelle.montanier@ch-forez.fr

Luc, MILLOT, CH du Forez, Montbrison, France, luc.millot@ch-forez.fr

Françoise, CREPET, CH du Forez, Montbrison, France, francoise.crepet@ch-forez.fr

Danielle, RATSIMBA, CH du Forez, Montbrison, France, danielle.ratsimba@ch-forez.fr

Kevin, BOUILLER, CHU JEAN MINJOZ, Besancon, France, kbouiller@chu-besancon.fr

Sophie, BOROT, CHU JEAN MINJOZ, Besancon, France, sophie.borot@univ-fcomte.fr

Isabelle, BRUCKERT, CHU JEAN MINJOZ, Besancon, France, ibruckert@chu-besancon.fr

Annie, CLERGEOT, CHU JEAN MINJOZ, Besancon, France, aclergeot@chu-besancon.fr

Franck, SCHILLO, CHU JEAN MINJOZ, Besancon, France, fschillo@chu-besancon.fr

Dorothée, VIGNES, CHU Antoine Béclère, Clamart, France, dorothée.vignes@aphp.fr

Muriel, BOURGEON-GHITTORI, CHU Antoine Béclère, Clamart, France, muriel.bourgeon@aphp.fr

Hamoud, LACHGAR, CHU Antoine Béclère, Clamart, France, hamoud.lachgar@aphp.fr

Claire, LAMBERT DE CURSAY, CHU Antoine Béclère, Clamart, France, claire.lambertdecursay@aphp.fr

Stéphane, LEVANTE, CHU Antoine Béclère, Clamart, France, stephane.levante@aphp.fr

Jean Charles, AUREGAN, CHU Antoine Béclère, Clamart, France, jean-charles.auregan@aphp.fr

Antoine, MERLET, CH Bretagne Atlantique, Vannes, France, antoine.merlet@ch-bretagne-atlantique.fr

Cécile, ZARAGOZA, CH Bretagne Atlantique, Vannes, France, cecile.zaragora@ch-bretagne-atlantique.fr

Gwénaëlle, ARNAULT, CH Bretagne Atlantique, Vannes, France, gwenaelle.arnault@ch-bretagne-atlantique.fr

Anne-Gaëlle, LE LOUPP, CH Bretagne Atlantique, Vannes, France, anne-gaelle-le8loupp@ch-bretagne-atlantique.fr

Olivier, LESIEUR, Hopital Saint Louis, La Rochelle, France, olivier.lesieur@ch-larochelle.fr

Mariam, RONCATO-SABERAN, Hopital Saint Louis, La Rochelle, France, mariam.roncato@ch-larochelle.fr

Didier, GOUET, Hopital Saint Louis, La Rochelle, France, didier.gouet@ch-larochelle.fr

Romain, LEMARIE, Hopital Saint Louis, La Rochelle, France, romain.lemarie@ch-larochelle.fr

Hong An, ALLANO, Hopital Saint Louis, La Rochelle, France, hong-an.allano@ght-atlantique17.fr

Emmanuel, VIVIER, Saint-Joseph Saint-Luc, Lyon, France, evivier@ch-stjoseph-stluc-lyon.fr

Caroline, PARISET, Saint-Joseph Saint-Luc, Lyon, France, cpariset@ch-stjoseph-stluc-lyon.fr

Cédric, LUYTON, Saint-Joseph Saint-Luc, Lyon, France, cluyton@ch-stjoseph-stluc-lyon.fr

Lucien, MARCHAND, Saint-Joseph Saint-Luc, Lyon, France, lmarchand@ch-stjoseph-stluc-lyon.fr

Fanny, DOROSZEWSKI, Saint-Joseph Saint-Luc, LYON, France, fdoroszewski@ch-stjoseph-stluc-lyon.fr

Matthieu, PECQUET, Saint-Joseph Saint-Luc, Lyon, France, mpecquet@ch-stjoseph-stluc-lyon.fr

Laurent, PERARD, Saint-Joseph Saint-Luc, Lyon, France, lperard@ch-stjoseph-stluc-lyon.fr

Sylvie, VUILLERMOZ-BLAS, Saint-Joseph Saint-Luc, Lyon, France, svuillermoz@ch-stjoseph-stluc-lyon.fr

Nicolas, KACKI, CHD de Vendée, La Roche Sur Yon, France, nicolas.kacki@chd-vendée.fr

Patricia, CHARRIER, CHD de Vendée, La Roche Sur Yon, France, patricia.charrier@chd-vendée.fr

Amélie, DUCET-BOIFFARD, CHD de Vendée, La Roche Sur Yon, France, amelie.ducet-boiffard@chd-vendee.fr

Françoise, DESROYS DU ROURE, CHD de Vendée, La Roche Sur Yon, France, francois.desroysduroure@chd-vendee.fr

Olivier, BOURRON, Pitié-Salpêtrière, Paris, France, olivier.bourron@aphp.fr

Dominique, BONNEFONT-ROUSSELOT, Pitié-Salpêtrière, Paris, France, dominique.rousselot@aphp.fr

Suzanne, LAROCHE, Pitié-Salpêtrière, Paris, France, Suzanne.laroche@aphp.fr

Franck, PHAN, Pitié-Salpêtrière, Paris, France, franck.phan@aphp.fr

Agnès, HARTEMANN, Pitié-Salpêtrière, Paris, France, agnes.hartemann@aphp.fr

Cyrielle, CAUSSY, CHU LYON SUD, Pierre Benite, France, cyrielle.caussy@chu-lyon.fr

Emmanuel, DISSE, CHU LYON SUD, Pierre Benite, France, emmanuel.disse@chu-lyon.fr

Emilie, BLOND, CHU LYON SUD, Pierre Benite, France, emilie.blond@chu-lyon.fr

Claude, GUERIN, Hôpital Croix Rousse, Lyon, France, claude.guerin@chu-lyon.fr

Thomas, PERPOINT, Hôpital Croix Rousse, Lyon, France, thomas.perpoint@chu-lyon.fr

Philippe, MOULIN, Hôpital Louis pradel, Lyon, France, philippe.moulin@chu-lyon.fr

Régine, CARTIER, Hôpital Louis pradel, Lyon, France, regine.cartier@chu-lyon.fr

Geoffroy, HARIRI, Hôpital Saint-Antoine, Paris, France, geoffroy.hariri@aphp.fr

Dorothée, CHOPIN, Hôpital Saint-Antoine, Paris, France, dorothee.chopin@aphp.fr

Camille, VATIER, Hôpital Saint-Antoine, Paris, France, camille.vatier@aphp.fr

Nathalie, BOURCIGAUX, Hôpital Saint-Antoine, Paris, France, nathalie.bourcigaux@aphp.fr

Emmanuelle, CHAIGNEAU, Hôpital Saint-Antoine, Paris, France, emmanuelle.chaigneau@aphp.fr

Sophie, CHRISTIN-MAITRE, Hôpital Saint-Antoine, Paris, France, sophie.christin-maitre@aphp.fr

Bruno, DONADILLE, Hôpital Saint-Antoine, Paris, France, bruno.donadille@aphp.fr

Bruno, FEVE, Hôpital Saint-Antoine, Paris, France, bruno.feve@aphp.fr

Sophie, LAMOTHE, Hôpital Saint-Antoine, Paris, France, sophie.lamothe@aphp.fr

Julie, SARFATI, Hôpital Saint-Antoine, Paris, France, julie.sarfati@aphp.fr

Pascal, PERNET, Hôpital Saint-Antoine, Paris, France, pascal.pernet@aphp.fr

Anne, CHAMBON, CH Côtes Basques, Bayonne, France, achambon@ch-cotebasque.fr

Delphine, DEMARSY, CH Côtes Basques, Bayonne, France, ddemarsy@ch-cotebasque.fr

Hugo, CAMPAGNE, CH Côtes Basques, Bayonne, France, hcampagne@ch-cotebasque.fr

Françoise, LATIL-PLAT, CH Avignon, Avignon, France, fplat@ch-avignon.fr

Monica, BERNE, CH Avignon, Avignon, France, MBeyrne@ch-avignon.fr

Marilyne, GRINAND, CH Avignon, Avignon, France, GRINAND.Marilyne@ch-avignon.fr

Marion, TOUZET, CH Avignon, Avignon, France, TOUZET.Marion@ch-avignon.fr

Aydrey, ZABULON, CHU Martinique, Fort de France, France, audrey.zabulon@chu-martinique.fr

Jocelyne, CRASPAG, CHU Martinique, Fort de France, France, jocelyne.craspag@chu-martinique.fr

Catherine, LEDOUX, CHU Martinique, Fort de France, France, catherine.ledoux@chu-martinique.fr

Cedric, CONTARET, CHU Martinique, Fort de France, France, Cedric.CONTARET@chu-martinique.fr

Blandine, JANAND-DELENNE, CH du Pays d'Aix, Aix en Provence, France, bdelenne@ch-aix.fr

Anaïs, GIRAUD, CH du Pays d'Aix, Aix en Provence, France, agiraud@ch-aix.fr

Marie Lou, LACRIMINI, CH du Pays d'Aix, Aix en Provence, France, mlacrimini@ch-aix.fr

Joëlle, ARRIVIE, CH de Bigorre, Tarbes, France, jarrivie@ch-tarbes-vic.fr

Deborah, ANCELLE, CH Le Havre, Le Havre, France, deborah.ancelle@ch-havre.fr

Carine, GUILLOIS, CH Le Havre, Le Havre, France, carine.guillois@ch-havre.fr

Bénédicte, FREMY, CH Agen, Agen, France, fremyb@ch-agen-nerac.fr

Amina, CHAALAL, CH Agen, Agen, France, chaalalam@ch-agen-nerac.fr

Gaëlle, BARRANDE, CH Argenteuil, Argenteuil, France, gaelle.barrande@ch-argenteuil.fr

Anne, DORANGE, CH Le Mans, Le Mans, France, adorange@ch-lemans.fr

Eglantine, ROUANET, CH Le Mans, Le Mans, France, erouanet@ch-lemans.fr

Dominique, SERET-BEGUE, CH Gonesse, Gonesse, France, dominique.seret-begue@ch-gonesse.fr

Audrey, SAOUD, CH Gonesse, Gonesse, France, audrey.saoud@ch-gonesse.fr

Anne-Marie, GUEDJ, CH Nîmes, Nîmes, France, anne.marie.guedj@chu-nimes.fr

Nathalie, BEDOS, CH Nîmes, Nîmes, France, nathalie.bedos@chu-nimes.fr

Fritz-Line, VELAYOUDOM, CHU Guadeloupe, Les Abymes, France, fritz-line.velayoudom@univ-antilles.fr

Marie, DUMAS, Hôpital St Vincent de Paul, Lille, France, dumas.marie@ghicl.net

Benoite, GONDA, Hôpital St Vincent de Paul, Lille, France, Gonda.Benoite@ghicl.net

Christine, COFFIN, CH Perigueux, Perigueux, France, christine.coffin@ch-perigueux.fr

Stéphanie, GIBIAT, CH Perigueux, Perigueux, France, urc@ch-perigueux.fr

Myriam, LUNGO, CH de Bastia, Bastia, France, myriam.lungo@gmail.com

Chantal, BULLY, Les Portes du Sud, Venissieux, France, C.BULLY@LESPORTESDUSUD.NET

Pierre, SERUSCLAT, Les Portes du Sud, Venissieux, France, p.serusclat@lesportesdusud.net

Stella, BULLY, Les Portes du Sud, Venissieux, France, stellabully.ecsel@gmail.com

Patricia, CARRE, Les Portes du Sud, Venissieux, France, patcarre69@gmail.com

Jean-Philippe, LEBERRE, Medipôle Hôpital Mutualiste, Villeurbanne, France, j.leberre@resamut.fr

Carlos, ELKHOURY, Medipôle Hôpital Mutualiste, Villeurbanne, France, c.elkhoury@resamut.fr

Marine, THIEUX, Medipôle Hôpital Mutualiste, Villeurbanne, France, m.thieux@resamut.fr

Laetitia, PARADISI-PRIEUR, Medipôle Hôpital Mutualiste, Villeurbanne, France, l.paradisi-prieur@resamut.fr
